# Prefrontal Regulation of Safety Learning during Ethologically Relevant Thermal Threat

**DOI:** 10.1523/ENEURO.0140-23.2024

**Published:** 2024-02-09

**Authors:** Ada C. Felix-Ortiz, Jaelyn M. Terrell, Carolina Gonzalez, Hope D. Msengi, Miranda B. Boggan, Angelica R. Ramos, Gabrielle Magalhães, Anthony Burgos-Robles

**Affiliations:** ^1^Department of Neuroscience, Developmental, and Regenerative Biology, The University of Texas at San Antonio, San Antonio, Texas 78249; ^2^Department of Psychological and Brain Sciences, Boston University, Boston, Massachusetts 02215; ^3^Brain Health Consortium, The University of Texas at San Antonio, San Antonio, Texas 78249

**Keywords:** behavioral flexibility, cingulate cortex, conflict, fear, seeking, social isolation

## Abstract

Learning and adaptation during sources of threat and safety are critical mechanisms for survival. The prelimbic (PL) and infralimbic (IL) subregions of the medial prefrontal cortex (mPFC) have been broadly implicated in the processing of threat and safety. However, how these regions regulate threat and safety during naturalistic conditions involving thermal challenge still remains elusive. To examine this issue, we developed a novel paradigm in which adult mice learned that a particular zone that was identified with visuospatial cues was associated with either a noxious cold temperature (“threat zone”) or a pleasant warm temperature (“safety zone”). This led to the rapid development of avoidance behavior when the zone was paired with cold threat or approach behavior when the zone was paired with warm safety. During a long-term test without further thermal reinforcement, mice continued to exhibit robust avoidance or approach to the zone of interest, indicating that enduring spatial-based memories were formed to represent the thermal threat and thermal safety zones. Optogenetic experiments revealed that neural activity in PL and IL was not essential for establishing the memory for the threat zone. However, PL and IL activity bidirectionally regulated memory formation for the safety zone. While IL activity promoted safety memory during normal conditions, PL activity suppressed safety memory especially after a stress pretreatment. Therefore, a working model is proposed in which balanced activity between PL and IL is favorable for safety memory formation, whereas unbalanced activity between these brain regions is detrimental for safety memory after stress.

## Graphical abstract

**Figure:**
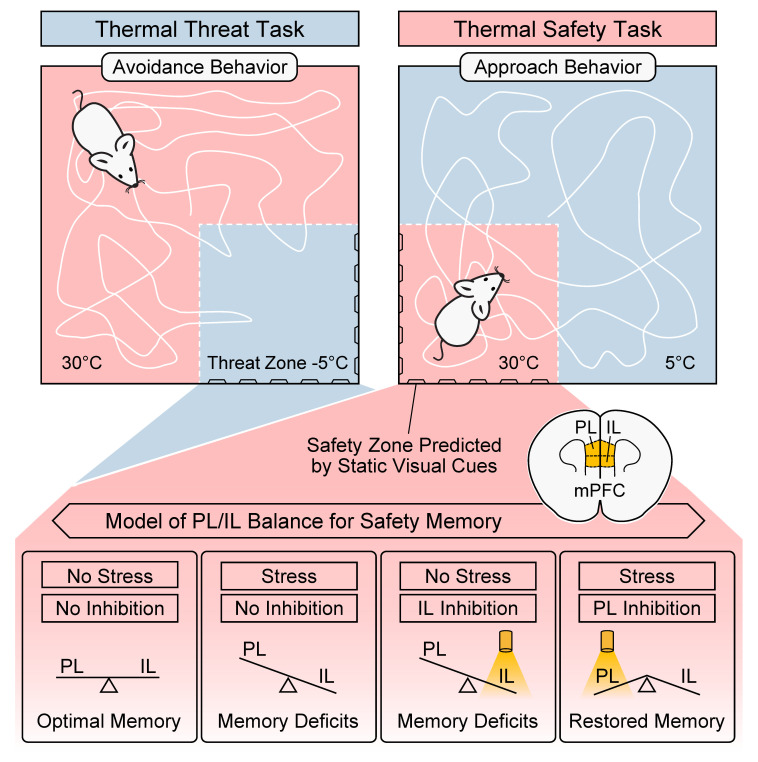
Visual Abstract

## Significance Statement

This study provides new insights on the role of prefrontal cortical processing for threat and safety learning during thermal challenge. For this, a novel behavioral paradigm was implemented in which laboratory mice learned that a particular spatial zone was associated with either a noxious cold temperature (“thermal threat”) or a pleasant warm temperature (“thermal safety”). Manipulations of neuronal activity revealed that the prelimbic and infralimbic subregions of the medial prefrontal cortex bidirectionally regulated memory formation for the thermal safety zone, but not for the thermal threat zone. In addition, the influence of these cortical regions during safety memory formation was altered when mice underwent a stress treatment to produce a disease-like state.

## Introduction

Threat and safety learning are fundamental mechanisms to promote behavioral adaptation, environmental fitness, survival, and emotional health. When the neural substrates involved in the processing of threat and safety are affected, for example, as a consequence of stress and emotional trauma, there is often an increase in the development of maladaptive states that are characterized by generalized fear, heightened anxiety, and impaired behavioral flexibility (Dunsmoor and Paz, 2015; Likhtik and Paz, 2015). Compelling evidence has been gathered over the years to describe in great details the neural mechanisms contributing to threat learning during health and disease (for review, see Maren and Quirk, 2004; Duvarci and Pare, 2014; Herry and Johansen, 2014; Tovote et al., 2015; LeDoux and Daw, 2018). However, the neural foundations supporting safety learning still remain elusive.

Growing evidence indicates that safety learning requires processing in neural networks that largely overlap with the ones for threat learning. At the front and center, there is the basolateral amygdala for dissociating cues that predict threat versus safety (Genud-Gabai et al., 2013; Sangha et al., 2013; Stujenske et al., 2014; Wen et al., 2022). Hippocampal areas are also crucial for encoding the contextual features that predict threat versus safety (Ji and Maren, 2005; Moscarello and Maren, 2018; Meyer et al., 2019). Great emphasis has also been given to the medial prefrontal cortex (mPFC) for integration of amygdala and hippocampal inputs to promote higher-order computations, decision-making, conflict resolution, and the eventual regulation of adaptive behavior depending on whether the environmental conditions predict threat or safety (Burgos-Robles et al., 2017; Corches et al., 2019; Bravo-Rivera and Sotres-Bayon, 2020; Fernandez-Leon et al., 2021; Tashjian et al., 2021).

The mPFC is divided into at least two major functional units, the dorsal and ventral divisions, with the prelimbic (PL) and infralimbic (IL) cortices as the most prominent areas controlling various mechanisms associated with threat and safety, respectively. For instance, PL activity promotes the acquisition and expression of conditioned responses such as freezing and avoidance when particular cues or contexts predict noxious outcomes such as electric shocks (Gilmartin and McEchron, 2005; Burgos-Robles et al., 2009, 2017; Sharpe and Killcross, 2014; Diehl et al., 2018; Capuzzo and Floresco, 2020; Twining et al., 2020; Jercog et al., 2021; Martin-Fernandez et al., 2023). In contrast, IL activity promotes the extinction and inhibition of freezing and avoidance when the cues or contexts no longer predict shock (Milad and Quirk, 2002; Vidal-Gonzalez et al., 2006; Burgos-Robles et al., 2007; Santini et al., 2008; Judo et al., 2010; Sierra-Mercado et al., 2011; Bravo-Rivera et al., 2014; Tao et al., 2021). Similar dissociations for PL and IL have been also reported during behavioral paradigms in which discrete contexts or cues explicitly predict threat versus safety (e.g., conditioned inhibition or cue/context discrimination tasks; Corches et al., 2019; Sangha et al., 2014, 2020). Collectively, these observations highlight the importance of PL and IL as essential elements in the networks mediating the processing of threat and safety signals to promote learning and bidirectional control of defensive behavior.

Notably, a lot of the fundamental knowledge on the mechanisms underlying threat and safety learning has been gathered using behavioral paradigms that involve electric shock punishment or its omission. Yet, how threat and safety learning are supported during more naturalistic conditions remain largely underexplored. Scientific interest has been emerging on how thermal stimuli serve as natural reinforcers for learning and behavioral adaptation. For instance, exposure to thermally noxious environments (i.e., with significantly cold or hot temperatures) typically elicits escape and avoidance behavior, whereas exposure to thermally pleasant or neutral environments (i.e., with temperatures closer to body core levels) typically promotes approach and seeking behavior (Wessnitzer et al., 2008; Ofstad et al., 2011; Ezzatpanah et al., 2021; Kanai et al., 2022). While these observations have been often emphasized from the standpoint of thermoregulatory behavior (Bokiniec et al., 2018; Nakamura, 2018), how thermal reinforcers serve for deeming environments as threatening or safe remains unexploited.

In the present study, we developed a new behavioral paradigm in which mice learned spatio-thermal contingencies for threat and safety. We then dissected the functional role of the PL and IL subregions of the mPFC using optogenetic-mediated silencing of glutamatergic principal neurons. We finally implemented a stress procedure to determine how PL and IL function during thermal threat and safety learning get affected during disease-related states.

## Materials and Methods

### Subjects

All procedures were approved by the Institutional Animal Care and Use Committee in compliance with the U.S. Public Health Service's Policy on Humane Care and Use of Laboratory Animals (PHS policy), the Guide for the Care and Use of Laboratory Animals, and the Society's Policies on the Use of Animals in Neuroscience Research. A total of 376 C57BL/6J adult mice were included in this study. They were acquired through a commercial supplier (Jackson Laboratory) and were ∼8–9 weeks of age on arrival at the vivarium. Upon arrival, mice were housed in polycarbonate homecages (four per cage) within a biocontainment rack with controlled temperature and pressure, and a 12-h light/dark cycle with lights on at 7:00 A.M. Mice were allowed to acclimate to the vivarium and group housing conditions for at least 2 weeks and underwent at least one handling session for ∼10 min prior to any procedure. Food and water were available *ad libitum* at all times, except during behavioral testing. In most of the study, only male mice were considered to reduce the number of variables that could potentially influence the results (Krueger and Sangha, 2021), which primarily relied on a novel behavioral paradigm to evaluate the learning of spatio-thermal contingencies for threat and safety for which previous literature is lacking. Nonetheless, one experiment was conducted in males versus females to examine possible sex differences in the impact of stress during the learning of spatio-thermal safety.

### Stereotaxic surgery

Craniotomies were performed for bilateral delivery of viral vectors and chronic implantation of optical fibers for optogenetic manipulations. All surgical procedures were performed under aseptic conditions using stereotaxic frames (KOPF), isoflurane anesthesia systems (Harvard Apparatus), digital temperature controllers (KOPF), and stereoscopes for magnification (AmScope). Prior to skin incision, pre-emptive analgesia was achieved with local injections of lidocaine and bupivacaine (7–8 mg/kg, each), followed by a subcutaneous injection of sustained-release meloxicam (Melox-SR, 4 mg/kg) to provide analgesia for at least 72 h. For selective PL targeting, viral vectors were infused in the following stereotaxic coordinates: +1.70 mm A/P, ±0.35 mm M/L, and −2.40 mm D/V, relative to bregma. Optical fibers were then positioned in the dorsal PL using the following coordinates: +1.70 mm A/P, ±0.75 mm M/L, −1.95 mm D/V, and a 10° M/L angle, relative to bregma. For selective IL targeting, viral vectors were infused in the following stereotaxic coordinates: +1.73 mm A/P, ±0.35 mm M/L, and −2.95 mm D/V, relative to bregma. Optical fibers were then positioned in the dorsal IL using the following coordinates: +1.73 mm A/P, ±1.25 mm M/L, −2.30 mm D/V, and a 20° M/L angle, relative to bregma. Optical implants were secured to the skull using a biocompatible adhesive containing methacrylate resin and 4-META monomer (C&B Metabond, Parkell), and self-cure orthodontic black resin acrylic (Ortho-Jet, Lang Dental). Incisions were sutured, mice were then transferred to a clean cage with a temperature-regulated floor, and *ad libitum* access to water and a soft food option (e.g., nutritional gel cup). After several hours, mice were transferred to the vivarium for full recovery. Postoperative care was provided as needed, using Melox-SR for pain and normal saline Ringer’s for dehydration.

### Optogenetic-mediated inhibition

After surgical procedures, a period of 8–12 weeks was allowed for efficient expression of viral vectors. After this period, mice underwent stress exposure or behavioral testing. Viral aliquots were obtained from commercial suppliers (Addgene and UNC Vector Core). Viral vectors were infused using a microsyringe pump system (UMP3T-2; NanoFil with 33 Ga needles; World Precision Instruments). A volume of 400 nl of the viral-containing medium was infused in each hemisphere at the target sites, at a rate of 100 nl/min. The infusion needles were kept at the target site for an additional 10 min to allow proper diffusion of viruses. Needles were then slowly withdrawn to prevent back pressure and leakage outside the target sites. For optogenetic-mediated neuronal silencing (“photoinhibition”), several cohorts of mice were transduced in the target areas with serotype-5 adeno-associated viral vectors coding for the outward proton-pump *Halorubrum sodomense* archaerhodopsin, fused to enhanced yellow fluorescent protein, under the calcium/calmodulin-dependent protein kinase-II alpha promoter (AAV_5_-CaMKllα-ArchT3.0-eYFP). For comparison purposes, other control cohorts were transduced with viral vectors that only encoded for eYFP (AAV_5_-CaMKIIα-eYFP). The optical fiber implants were constructed in-house and consisted of Ø300 µm multimode cores (NA = 0.39; Thorlabs) attached to stainless steel ferrules (Ø1.25 mm OD, 330 µm ID bore, Precision Fiber Products). The ferrule ends were polished until achieving a light transmission efficiency >85%, using a fiber connector micropolisher (SpecPro, KrellTech). Prior to behavioral testing, mice were acclimated to being connected to fiber-optic patch cords (branching fibers; Ø200 µm cores; NA = 0.22; Doric Lenses), which were attached to optical rotaries (FRJ 1 × 1 FC/PC, Doric Lenses), which in turn were connected to red-shifted DPSS lasers (200 mW max, 589 nm, Opto Engine). Laser output was moderated by mechanical laser beam shutters (100 Hz SR475 Shutter Heads, SRS), which were controlled with TTL pulses using a hardware/software optogenetic interface. During behavioral experiments, photoinhibition was performed using constant delivery of laser light. Laser power was set to <5 mW at the tip of the patch cords.

### Social isolation stress

Social isolation was used to examine the impact of stress on the learning of spatio-thermal contingencies for threat and safety. While controls always remained in group-housing conditions, mice in the stress groups underwent individual housing for 12 d. After this isolation period, the stressed mice were returned to group-housing conditions with their former cagemates for a period of 2 weeks prior to behavioral testing. During stress, all mice were housed in the same room and biocontainment rack within the vivarium, and bodyweights were measured every other day to evaluate changes in weight gain. In addition, baseline bodyweight prior to stress was used in a post hoc analysis to assign social ranks within individual homecages (i.e., dominant mice tend to be larger and heavier than subordinates; Fulenwider et al., 2022) and evaluate the possible contribution of social status in behavior and stress vulnerability (i.e., dominance may confer stress resiliency; LeClair et al., 2021).

### Thermal threat task

For this novel task, a square-shaped acrylic box (i.e., open-field; 30 × 30 × 30 cm) was prepared in which distinct parts of the floor had different temperatures. While one quadrant had a noxious cold temperature (−5°C, “threat zone”; achieved by placing a mixture of regular and dry ice underneath the acrylic floor), the other quadrants were maintained at a level closer to core body temperature (30°C; achieved by placing warming mats underneath the acrylic floor). The stability of these temperatures was frequently monitored using a temperature-sensing infrared camera (FLIR C5). To facilitate spatial orientation and the formation of a spatial-based memory that represented the threat zone, visual cues were added to the walls of the box to differentiate the threat zone (with plus symbols) from the other zones (vertical bars). These proximal visual cues, as well as other distal cues within the procedure room, were maintained consistent across sessions and across the entire study to facilitate the comparison of findings. Furthermore, the box was always positioned on the same table while maintaining the same orientation across sessions. Yet, the orientation of the box was counterbalanced across mice (i.e., for some mice the threat zone was facing north and for other mice the threat zone was facing south). For a particular experiment, the box was rotated 180° during one of the sessions to validate the idea of spatial orientation and learning. During the training session, which lasted 10 min, mice were placed within the quadrant that was directly across from the threat zone. Twenty-four hours later during a recall session, which lasted 3 min, mice were placed again within the quadrant that was directly across from the zone that predicted threat, but the thermal reinforcer (−5°C) was not included to properly evaluate long-term memory for the threat zone. Recall of threat memory was implied from enduring avoidance behavior (i.e., lack of time spent within the zone that predicted threat) despite the absence of the cold stimulus. During optogenetic manipulations, the laser treatment was performed during the entire duration of the training session, but not during recall.

### Thermal safety task

This version of the task was conducted in different groups of mice to examine spatial-based safety learning. For this, the square-shaped acrylic box (30 × 30 × 30 cm) now had one quadrant with a pleasant warm temperature (30°C, “safety zone”), whereas the other quadrants had an uncomfortable temperature (5°C). This time, the cold zones were kept above freezing levels (i.e., >0°C) to persuade mice to explore the entire arena while reducing autonomous reflexes associated with physical pain. Yet, it has been reported that temperatures lower than 12°C are still perceived as significantly noxious (Lewis and Griffith, 2022). To facilitate spatial orientation and the formation of a spatial-based memory that represented the safety zone, visual cues were added to the walls of the box to differentiate the safety zone (with plus symbols) from the other zones (vertical bars). These proximal visual cues, as well as other distal cues within the procedure room, were maintained consistent across sessions and experiments and were exactly the same as the ones used during the thermal threat task to facilitate comparisons of findings across the study. Furthermore, the box was always positioned on the same table with the same orientation across sessions. Yet, the orientation of the box was counterbalanced across mice (i.e., for some mice the safety zone was facing north and for other mice the safety zone was facing south). For a particular experiment, the box was rotated 180° during one of the sessions to validate the idea of spatial orientation and learning. During the training session, which lasted 10 min, mice were placed within the quadrant that was directly across from the safety zone. Twenty-four hours later during a recall session, which lasted 3 min, mice were placed again within the quadrant that was directly across from the zone that predicted safety, but the thermal reinforcer (30°C) was not included to properly evaluate long-term memory for the safety zone. Recall of the safety memory was implied from enduring approach behavior (i.e., time spent within the zone that predicted safety) despite the absence of the warm stimulus. During optogenetic manipulations, the laser treatment was performed during the entire duration of the training session, but not during recall.

### Elevated-plus maze

The elevated-plus maze assay was used to evaluate anxiety-like behavior. The plus maze apparatus consisted of two open arms (30 × 5 cm) and two close arms (30 × 5 × 15 cm) that intersected at a central zone (5 × 5 cm) and were elevated from a tabletop (40 cm). This apparatus was acquired through a commercial source (San Diego Instruments) and was made out of a beige-colored ABS plastic material for enhanced contrast with mice. This assay lasted for a total of 9 min and was conducted in a room with moderate lighting levels. For optogenetic experiments, the session was divided into three 3 min epochs with alternating laser status (Off-On-Off). Video capturing was performed to track the position of animals within the apparatus using automated software. The total time that mice spent in the open arms versus the closed arms was quantified during the different laser epochs. Mice typically avoid the open arms, which are more anxiogenic (Walf and Frye, 2007).

### Open-field test

The open-field test assay was used to examine anxiety-like behavior and general locomotion (Seibenhener and Wooten, 2015). Mice were allowed to freely explore a square-shaped arena (40 × 40 × 40 cm) for a total of 9 min. To examine anxiety, the arena was virtually divided into a center zone (25 × 25 cm) and an outer periphery zone. During optogenetic manipulations, the session was divided into three 3 min epochs with alternating laser status (Off-On-Off). The time that mice spent in the center zone was evaluated as an indicator of anxiety-like behavior (i.e., the center zone is anxiogenic whereas the periphery is safer). To examine locomotor activity, quantifications were made for the speed of movement and total distance traveled by mice during the different epochs.

### Real-time place preference or aversion

During this assay, mice were allowed to freely explore a rectangular acrylic box (50 × 30 × 22 cm) that was virtually divided into two halves with alternated optogenetic manipulation (Bimpisidis et al., 2020). That is, mouse entry into one half of the arena resulted in closed-loop photoinhibition treatment (i.e., Laser-On), whereas mouse entry into the other half of the arena resulted in the cessation of photoinhibition (i.e., Laser-Off). Two daily sessions lasting 30 min each were conducted for each mouse. Mice started out each session in the Laser-Off side. The Laser-Off and Laser-On sides were counterbalanced between the 2 d. The total time that mice spent on each treatment side was measured using automated animal tracking software. The difference score between the Laser-On and Laser-Off sides was then calculated for each mouse and plotted for group comparisons.

### Software and statistical analysis

ANY-maze software was used to capture videos of the behavioral sessions, control the laser shutters, and track mice for automated quantifications of behavior. Behavioral measurements included the following: (1) time that mice spent in distinct zones of the test arenas; (2) number of entries into the distinct zones; (3) head dipping into zones of interest; (4) total distance traveled in the arenas; (5) average speed of locomotion; and (6) freezing or immobilization behavior (Blanchard et al., 1986). Data was then transferred to MS Excel and GraphPad Prism for analysis, graphing, and statistical assessment. Normality of the data was verified using the Kolmogorov–Smirnov test, and results were plotted as mean ± standard error of the mean. Data from individual mice was also plotted as scattered dots over the mean plots. Statistical significance and group differences were evaluated using two-way repeated measures ANOVA and Bonferroni’s post hoc tests, otherwise indicated. Multiplicity adjusted *p* values were always considered to account for multiple comparisons. The chi-square test was used to compare proportions of mice. Significance thresholds were set to *p < 0.05, **p < 0.01, ***p < 0.001, or ****p < 0.0001.

### Histology

Mice were deeply anesthetized with isoflurane and transcardially perfused with ice-cold phosphate-buffered saline (1× PBS) and paraformaldehyde (4%-PFA). Brains were collected, fixed for 24 h in 4%-PFA, and then were equilibrated for 48 h in a 4%-PFA/30%-sucrose solution. Coronal sections were then cut at 40 µm using a sliding microtome (HM430, Thermo Scientific). Brain sections containing the mPFC regions of interest (PL and IL) were then mounted on superfrost microscope slides and immersed in a fluorescence-compatible mounting media containing DAPI (Fluoromount-G, SouthernBiotech). Images were then acquired using a fluorescence microscope with automated capturing and stitching capabilities (BX43, cellSens, Olympus). Viral vector expression and the location of optical fiber tips were then reconstructed in coronal drawings of the mPFC, based on a mouse brain atlas (Paxinos and Franklin, 2001). Only animals in which the PL and IL regions were targeted individually in both hemispheres were considered for analysis. The anatomical boundaries between the PL and IL subregions were determined based on anatomical features and transitions in the shape of cortical layers. Due to viral leakage or optical fiber misplacement, 36 mice were excluded from the study.

### Availability of materials and data

Data will be made available for scientific use upon request. Viral vectors and sequences are available through commercial sources (e.g., Addgene and UNC Vector Core).

## Results

### Spatial-based threat learning using a noxious cold thermal reinforcer

**Figure 1.**
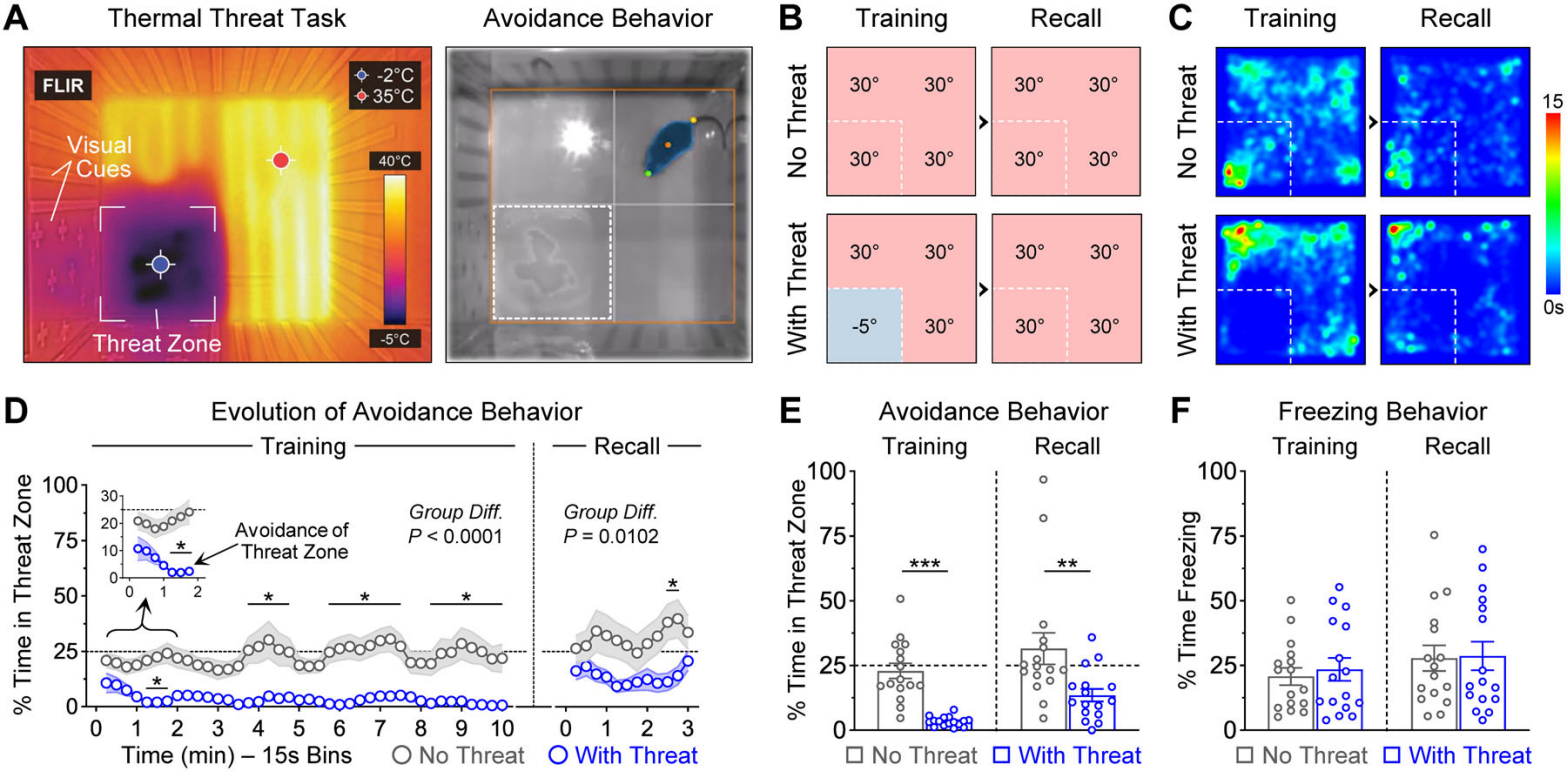
Spatial-based threat learning using a noxious cold temperature. ***A***, Pictures of the training arena at the infrared and visible spectra. In one quadrant, the floor had a noxious cold temperature (approximately −5°C; “Threat Zone”), whereas the other quadrants were kept closer to body core temperature (∼30°C). The cold threat zone was differentiated from the other zones using visuospatial cues on the walls of the apparatus (e.g., plus symbols vs vertical bars, respectively). ***B***, Experimental design to validate the threat task. During a 10 min training session, the control group never experienced the threat zone (“No Threat”, *N* = 16), whereas the experimental group experienced the threat zone which had a noxious temperature of −5°C (“With Threat”, *N* = 16). During a 3 min recall test the next day, both groups were exposed to the same apparatus but in the absence of the cold zone. ***C***, Group heatmaps based on animal location. The color scale represents time in seconds. ***D***, Evolution of avoidance behavior, assessed as the time spent in the threat zone. Inset shows a zoom into the first 2 min of training. These line plots were smoothed using a three-bin rolling average method. Asterisks (*) indicate the time bins in which group differences reached significances of at least *p *< 0.05. ***E***, Average time spent in the threat zone during each session. The experimental group exhibited robust avoidance of the threat zone during the training session (***p = 0.0006), as well as during the subsequent test session despite the lack of thermal reinforcer (**p = 0.0015). These findings indicate that the experimental group learned a spatial-based threat memory that guided defensive behavior during the recall test. ***F***, No significant differences were detected for freezing behavior, which remained relatively low throughout the task (Training, *p *= 0.90; Recall, *p *= 0.99). See Extended Data Figure 1-1 for additional behavioral measurements during the thermal threat task and Extended Data Figure 1-2 for an additional experiment that tested the contribution of the visuospatial cues on the walls of the apparatus for memory formation during the thermal threat task. (In this and subsequent figures, data is shown as mean ±  SEM, and the scattered dots that are superimposed on the bars represent data for individual mice. The dashed line at 25% represents the expected time of zone exploration by chance.)

The first goal of this study was to determine whether exposure to a novel environment containing a noxious cold temperature could produce spatial-based threat learning. For this, a square-shaped open field arena was designed with a particular quadrant having a significantly cold temperature (−5°C, “threat zone”), while the other quadrants were maintained at a comfortable temperature (30°C; Fig. 1*A*, left panel). To facilitate spatial-based threat learning, we added visual cues to the walls of the apparatus (e.g., plus symbols versus vertical columns) to differentiate the thermal threat zone from the other zones. During a 10 min training session, the most prominent behavior exhibited by mice was avoidance of the cold-paired threat zone (Fig. 1*A*, right panel and Multimedia Video 1).

10.1523/ENEURO.0140-23.2024.video.1 Multimedia Video 1. Representative behavior during the thermal threat task. A full ten-minute training session is shown in the video clip, running at 20x speed. The threat zone located at the bottom left corner had a noxious cold temperature (-5°C), whereas the other quadrants had a comfortable temperature (30°C). Visuospatial cues on the walls of the apparatus differentiated the threat zone from the rest of the arena (plus symbols versus vertical bars, respectively). Software was used for automated mouse tracking and quantification of behavior. The orange dot represents the body center, the green dot represents the nose point, and the yellow dot represents the tail point. Download Video 1, MP4 file.

10.1523/ENEURO.0140-23.2024.f1-1 Figure 1-1 Additional measurements during the thermal threat task. ***A,*** Distance to the threat zone, measured from the center of the mouse body to the center of the zone (Group, *F*_(1,30)_ = 2.77, *P* = 0.11; Time, *F*_(1,30)_ = 3.62, *P* = 0.067; Interaction, *F*_(1,30)_ = 3.88, *P* = 0.058; Training: *P = 0.034; Recall: *P* > 0.99). ***B,*** Entries into the threat zone (Group, *F*_(1,30)_ = 8.71, *P* = 0.006; Time, *F*_(1,30)_ = 0.15, *P* = 0.70; Interaction, *F*_(1,30)_ = 12.6, *P* = 0.0013; Training: ****P < 0.0001; Recall: *P* > 0.99). ***C,*** Head dipping into the threat zone (Group, *F*_(1,30)_ = 0.94, *P* = 0.34; Time, *F*_(1,30)_ = 13.3, *P* = 0.001; Interaction, *F*_(1,30)_ = 1.27, *P* = 0.27; Training: *P* = 0.30; Recall: *P* > 0.99). ***D,*** General locomotion in the arena (Group, *F*_(1,30)_ = 2.54, *P* = 0.12; Time, *F*_(1,30)_ = 5.48, *P* = 0.026; Interaction, *F*_(1,30)_ = 4.01, *P* = 0.054; Training: *P = 0.037; Recall: *P* > 0.99). [No Threat: N = 16, With Threat: N = 16]. Download Figure 1-1, TIF file.

**Figure 2.**
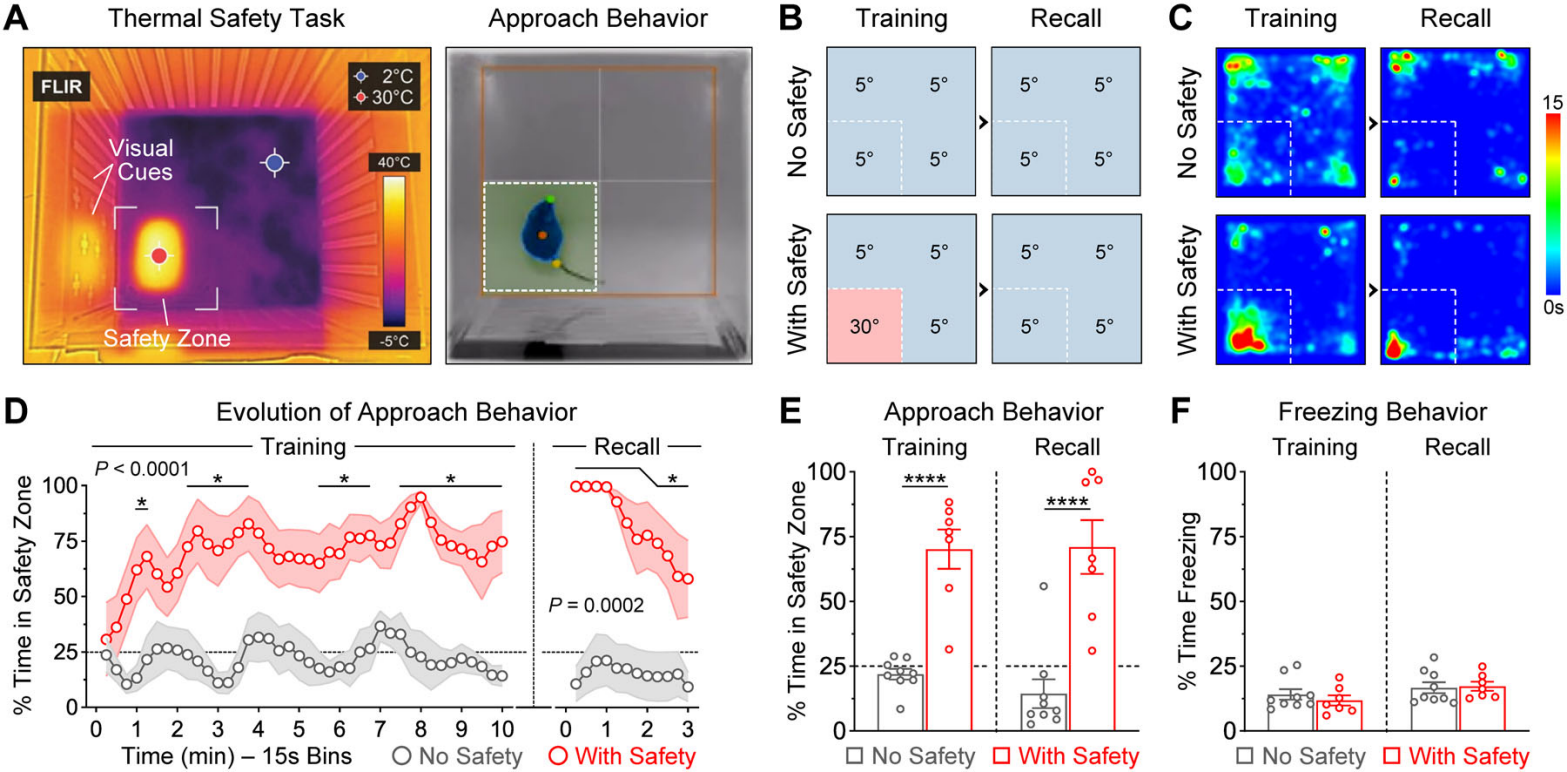
Spatial-based safety learning using a pleasant warm temperature. ***A***, Pictures of the training arena at the infrared and visible spectra. In this version of the task, one quadrant had a pleasant warm temperature (∼30°C; “Safety Zone”), whereas the other quadrants were kept at a thermally challenging level (∼5°C). The safety zone was differentiated from the other zones using visuospatial cues on the walls of the apparatus (e.g., plus symbols vs vertical bars, respectively). ***B***, Experimental design to validate the safety task. During a 10 min training session, the control group never experienced the safety zone (“No Safety”, *N* = 9), whereas the experimental group experienced the safety zone which had a pleasant temperature of 30°C (“With Safety”, *N* = 7). During a 3 min recall test the next day, both groups were exposed to the same apparatus but in the absence of the warm zone. ***C***, Group heatmaps based on animal location. The color scale represents time in seconds. ***D***, Evolution of approach behavior, assessed as the time spent in the safety zone. These line plots were smoothed using a three-bin rolling average method. Asterisks (*) indicate the time bins in which group differences reached significances of at least *p *< 0.05. ***E***, Average time spent in the safety zone during each session. The experimental group exhibited robust approach behavior to the safety zone during both training and the recall test despite the lack of the thermal reinforcer (****p < 0.0001). These findings indicate that the experimental group learned a spatial-based safety memory that guided defensive behavior during the recall test. ***F***, No significant differences were detected in freezing behavior, which remained relatively low throughout this task (Training, *p *= 0.87; Recall, *p *> 0.99). See Extended Data Figure 2-1 for additional behavioral measurements during the thermal safety task and Extended Data Figure 2-2 for an additional experiment that tested the contribution of the visuospatial cues on the walls of the apparatus for memory formation during the thermal safety task.

10.1523/ENEURO.0140-23.2024.f1-2 Figure 1-2 Visuospatial cues contributed to memory formation during the thermal threat task. ***A,*** Experimental design. For the experimental group, the box was rotated 180° during the recall test, so that the visual cues that predicted threat were now positioned somewhere else (No Rotation: N = 12, Rotated 180°: N = 12). ***B,*** Avoidance of the zone that predicted thermal threat, relative to the position of the visual cues. Similar to controls, the experimental group exhibited robust avoidance to the correct zone that predicted threat (Group, *F*_(1,22)_ = 1.24, *P* = 0.28; Time, *F*_(1,22)_ = 9.16, *P* = 0.0062; Interaction, *F*_(1,22)_ = 4.00, *P* = 0.058; Training, *P* > 0.99; Recall, *P* = 0.070). ***C,*** Avoidance of the opposite zone. Interestingly, the experimental group exhibited robust avoidance to this zone, which was the one associated with thermal threat during the training session. A significant group difference was detected during recall (Group, *F*_(1,22)_ = 7.37, *P* = 0.013; Time, *F*_(1,22)_ = 5.61, *P* = 0.027; Interaction, *F*_(1,22)_ = 10.6, *P* = 0.0037; Training, *P* > 0.99; Recall, ***P = 0.0002). ***D,*** A comparison of avoidance behavior during the recall test, considering the new location versus the old location of the visual cues that predicted the threat zone. The group with rotated cues exhibited similar levels of avoidance to the new and old locations (Paired T-test: *t*_(11)_ = 0.06, *P* = 0.96). Therefore, while these findings are consistent with the idea that proximal cues played an important role for the integration of spatial and thermal information to promote learning for the threat zone, these findings also suggest that distal cues (e.g., other visual elements within the procedure room) may have also played a significant role. Download Figure 1-2, TIF file.

To validate the thermal threat task, we made comparisons between two groups of male mice. The control group did not experience the threat zone during the training session (“No Threat”; Fig. 1*B*, top left panel). In contrast, the experimental group experienced the threat zone during training (“With Threat”; Fig. 1*B*, bottom left panel). The next day, both groups were tested in the same arena without further thermal reinforcer (Fig. 1*B*, right panels). The average cumulative time spent in the distinct zones of the arena is illustrated in the group heatmaps (Fig. 1*C*).

Significant group differences were detected in the time spent within the quadrant of interest (Fig. 1*D*,*E*). The control group exhibited ∼25% of time spent in the quadrant of interest during both sessions, which was indicative of exploratory behavior by chance. In contrast, the experimental group exhibited time-dependent development of avoidance behavior to the thermal threat zone during the training session and continued to exhibit this behavior during the long-term recall test even when the noxious cold temperature was no longer present. Two-way ANOVA tests revealed significant group differences during both sessions (Fig. 1*D*; Training Session: Group, *F*_(1,30) _= 43.4, *p *< 0.0001; Time Bins, *F*_(39,1170) _= 0.90, *p *= 0.64; Interaction, *F*_(39,1170) _= 1.16, *p *= 0.23; Recall Session: Group, *F*_(1,30) _= 7.52, *p *= 0.010; Time Bins, *F*_(11,330) _= 0.93, *p *= 0.51; Interaction, *F*_(11,330) _= 0.80, *p *= 0.64). Calculations of the averaged time spent within the quadrant of interest also revealed robust group differences (Fig. 1*E*; Group, *F*_(1,30) _= 26.3, *p *< 0.0001; Session, *F*_(1,30) _= 6.99, *p *= 0.013; Interaction, *F*_(1,30) _= 0.04, *p *= 0.84). Post hoc tests on the average data confirmed that the experimental group spent significantly less time in the zone of interest during both sessions, compared with the control group for the corresponding zone (Fig. 1*E*; *p *= 0.0006 during the training session, *p *= 0.0015 during the recall session). Additional group differences were detected in the distance to the threat zone and entries into the threat zone (Extended Data Fig. 1-1). No differences were noted in freezing behavior (Fig. 1*F*: Group, *F*_(1,30) _= 0.04, *p *= 0.84; Session, *F*_(1,30) _= 44.4, *p *< 0.0001; Interaction, *F*_(1,30) _= 0.00, *p *= 0.99), which is often associated with threat learning (Blanchard et al., 1986) but appeared to be a marginal behavioral factor in the present task. Therefore, spatial threat learning and memory were achieved using cold thermal reinforcement, and this type of learning was characterized by strong development of adaptive avoidance behavior to the cold zone.

To further validate the thermal threat task, we examined the role of the visuospatial cues provided on the walls of the box to facilitate spatial orientation, discrimination, and learning for the threat zone. To do this, a new cohort of male mice underwent training in the threat task, and the next day half of the mice were tested with the visual cues rotated 180° relative to their position during training (Extended Data Fig. 1-2*A*). Consistent with the idea that proximal visual cues would facilitate learning, the group of mice with rotated cues exhibited strong avoidance behavior to the new zone containing the cues that predicted thermal threat (Panel 1-2B). Interestingly, this group also continued to exhibit avoidance to the old zone that predicted thermal threat (Panel 1-2C). No significant difference was detected when comparing the avoidance levels during recall for the new zone versus the old zone in the group with rotated cues (Panel 1-2D). Thus, while the proximal cues played an important role for the establishment of the spatial memory that represented thermal threat, it is possible that distal cues also played a role (Hébert et al., 2017).

### Spatial-based safety learning using a pleasant warm thermal reinforcer

The next goal of this study was to determine whether spatial-based safety learning could also be achieved in a similar way to thermal threat learning, but using a pleasant thermal reinforcer. For this, we modified the square-shaped open field arena to have one quadrant associated with a pleasant warm temperature (30°C, “safety zone”), whereas the temperature in the other quadrants was dropped to a significantly lower level (5°C), thereby persuading mice to seek safety within a particular zone with discrete visual cues (Fig. 2*A*, left panel). After some initial exploration of the entire arena, the most prominent behavioral response exhibited by mice during this task was approach to the zone containing the pleasant warm temperature (Fig. 2*A*, right panel and Multimedia Video 2).

10.1523/ENEURO.0140-23.2024.video.2 Multimedia Video 2. Representative behavior during the thermal safety task. A full ten-minute training session is shown at 20x speed. The safety zone located at the bottom left corner had a pleasant warm temperature (30°C), whereas the other quadrants had an uncomfortable temperature (5°C). During this version of the task, visuospatial cues now differentiated the safety zone from the rest of the arena (plus symbols versus vertical bars, respectively). Software was used for automated mouse tracking and quantification of behavior. The orange dot represents the body center, the green dot represents the nose point, and the yellow dot represents the tail point. Download Video 2, MP4 file.

10.1523/ENEURO.0140-23.2024.f2-1 Figure 2-1 Additional measurements during the thermal safety task. ***A,*** Average distance to the safety zone, measured from the center of the mouse body to the center of the zone (Group, *F*_(1,14)_ = 59.9, *P* < 0.0001; Time, *F*_(1,14)_ = 9.19, *P* = 0.009; Interaction, *F*_(1,14)_ = 0.52, *P* = 0.48; Training: ***P = 0.0002; Recall: ****P < 0.0001). ***B,*** Entries into the safety zone (Group, *F*_(1,14)_ = 1.34, *P* = 0.27; Time, *F*_(1,14)_ = 14.9, *P* = 0.002; Interaction, *F*_(1,14)_ = 1.28, *P* = 0.27; Training: *P* = 0.23; Recall: *P* > 0.99). ***C,*** Head dipping into the safety zone (Group, *F*_(1,14)_ = 6.46, *P* = 0.024; Time, *F*_(1,14)_ = 34.0, *P* < 0.0001; Interaction, *F*_(1,14)_ = 14.0, *P* = 0.002; Training: ***P = 0.0004; Recall: *P* > 0.99). ***D,*** General locomotion in the arena (Group, *F*_(1,14)_ = 6.29, *P* = 0.025; Time, *F*_(1,14)_ = 43.3, *P* < 0.0001; Interaction, *F*_(1,14)_ = 0.08, *P* = 0.78; Training: *P* = 0.074; Recall: *P* = 0.15). [No Safety: N = 9, With Threat: N = 7]. Download Figure 2-1, TIF file.

10.1523/ENEURO.0140-23.2024.f2-2 Figure 2-2 Visuospatial cues contributed to memory formation during the thermal threat task. ***A,*** Experimental design. For the experimental group, the box was rotated 180° during the recall test, so that the visual cues that predicted threat were now positioned somewhere else (No Rotation: N = 11, Rotated 180°: N = 10). ***B,*** Approach to the zone that predicted thermal safety, relative to the position of the visual cues. Similar to controls, the experimental group exhibited approach behavior to the zone that correctly predicted safety (Group, *F*_(1,19)_ = 0.36, *P* = 0.56; Time, *F*_(1,19)_ = 11.1, *P* = 0.0035; Interaction, *F*_(1,19)_ = 0.001, *P* = 0.97; Training, *P* > 0.99; Recall, *P* > 0.99). ***C,*** Approach to the opposite zone. Both groups exhibited low levels of approach behavior to this zone (Group, *F*_(1,19)_ = 1.20, *P* = 0.29; Time, *F*_(1,19)_ = 0.67, *P* = 0.42; Interaction, *F*_(1,19)_ = 1.11, *P* = 0.30; Training, *P* = 0.28; Recall, *P* > 0.99). ***D,*** A comparison of approach behavior during the recall test, considering the new location versus the old location of the visual cues that predicted the safety zone. The group with rotated cues exhibited significantly more approach to the new location (Paired T-test: *t*_(9)_ = 3.41, **P = 0.008). These findings are consistent with the idea that proximal visual cues played a prominent role for the integration of spatial and thermal information to promote learning for the safety zone. Download Figure 2-2, TIF file.

To validate the thermal safety task, we made comparisons between two groups of male mice. The control group did not experience the safety zone during the training session (“No Safety”; Fig. 2*B*, top left panel). In contrast, the experimental group experienced the safety zone during training (“With Threat”; Fig. 2*B*, bottom left panel). The next day, both groups were tested in the same arena without further thermal reinforcer (Fig. 2*B*, right panels). The average cumulative time that mice spent in the distinct zones of the arena is illustrated in the group heatmaps (Fig. 2*C*).

Significant group differences were detected in the time spent within the quadrant of interest (Fig. 2*D*,*E*). The control group exhibited ∼25% of time spent within the quadrant of interest during both sessions, which was indicative of exploratory behavior by chance. In contrast, the experimental group exhibited time-dependent development of approach behavior to the thermal safety zone during the training session and continued to exhibit this behavior during the recall test even when the pleasant warm temperature was no longer present. Two-way ANOVA tests revealed significant group differences during both sessions (Fig. 2*D*; Training Session: Group, *F*_(1,14) _= 47.1, *p *< 0.0001; Time Bins, *F*_(39,546) _= 1.33, *p *= 0.089; Interaction, *F*_(39,546) _= 0.96, *p *= 0.54; Recall Session: Group, *F*_(1,14) _= 25.2, *p *= 0.0002; Time Bins, *F*_(11,154) _= 4.25, *p *< 0.0001; Interaction, *F*_(11,154) _= 2.62, *p *= 0.0043). Calculations of the averaged time spent within the quadrant of interest also revealed robust group differences (Fig. 2*E*; Group, *F*_(1,14) _= 76.6, *p *< 0.0001; Session, *F*_(1,14) _= 0.23, *p *= 0.64; Interaction, *F*_(1,14) _= 0.35, *p *= 0.56). Post hoc tests confirmed that the experimental group spent significantly more time in the zone associated with thermal safety, relative to controls for the corresponding zone (*p *< 0.0001 during both sessions). Additional group differences were detected in some supplementary measurements, such as in the distance that mice maintained to the safety zone (Extended Data Fig. 2-1). No differences were noted in freezing behavior which remained low during this task (Fig. 2*F*: Group, *F*_(1,14) _= 0.05, *p *= 0.83; Session, *F*_(1,14) _= 39.0, *p *< 0.0001; Interaction, *F*_(1,14) _= 0.42, *p *= 0.53). Therefore, learning and memory for spatial safety was achieved using warm thermal reinforcement, and this type of learning was notably characterized by strong development of adaptive approach behavior to the warm zone.

To further validate the thermal safety task, we examined the role of the visuospatial cues provided on the walls of the box to facilitate spatial orientation, discrimination, and learning for the safety zone. A new cohort of male mice underwent training in the safety task, and the next day half of the mice were tested with the visual cues rotated 180° relative to their position during training (Extended Data Fig. 2-2*A*). Consistent with the idea that proximal visual cues would facilitate learning, the group of mice with rotated cues exhibited significantly more approach behavior to the new zone containing the visual cues that predicted thermal safety (Panels 2-2B, 2-2C, and 2-2D). Therefore, the proximal cues had a strong influence for the establishment of the spatial memory that represented thermal safety.

### Memory formation during thermal threat learning did not require prefrontal activity

**Figure 3.**
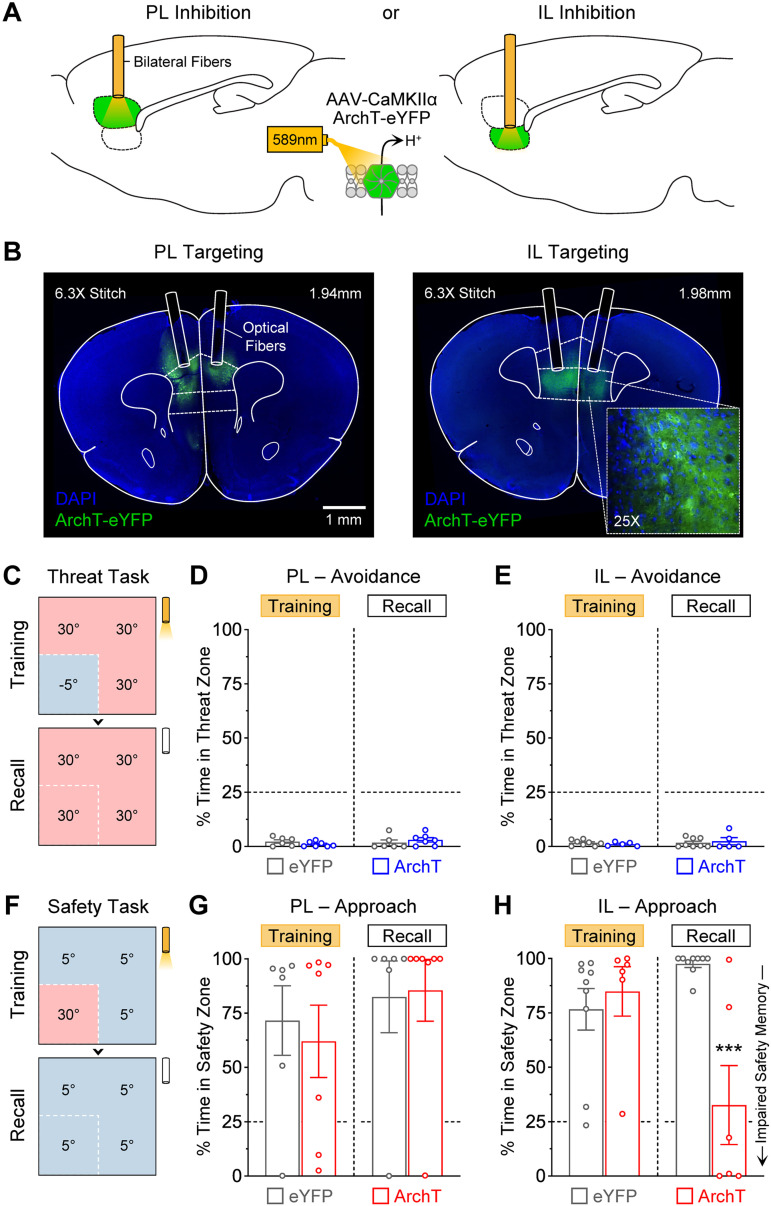
Selective photoinhibition of the IL but not PL subregion of the mPFC impaired the formation of safety memory. ***A***, Strategy to selectively inhibit the mPFC regions using a yellow light-driven proton pump (ArchT; archaerhodopsin). Controls only expressed a fluorophore (eYFP; enhanced yellow fluorescence protein). ***B***, Representative photomicrographs of PL and IL targeting with the optical fibers and viral vectors. ***C***, Photoinhibition was first performed during the thermal threat task. ***D***, PL inhibition did not affect memory formation for the threat zone (eYFP, *N* = 6; ArchT, *N* = 7; Training, *p *= 0.66; Recall, *p *= 0.64). ***E***, IL inhibition did not affect memory formation for the threat zone either (eYFP, *N* = 8; ArchT, *N* = 5; Training, *p *> 0.99; Recall, *p *> 0.99). ***F***, Photoinhibition was then performed in different cohorts during the thermal safety task. ***G***, PL inhibition did not affect memory formation for the safety zone (eYFP, *N* = 6; ArchT, *N* = 7; Training, *p *> 0.99; Recall, *p *> 0.99). ***H***, IL inhibition produced a robust impairment in memory formation for the safety zone without affecting performance during training (eYFP, *N* = 9; ArchT, *N* = 6; Training, *p *> 0.99; Recall, ***p = 0.003). See Extended Data Figure 3-1 for a full histological reconstruction of optical fiber placements and viral infusion sites and Extended Data Figure 3-2 for additional behavioral measurements during PL or IL inhibition.

Our next goal was to examine potential contributions of the PL and IL subregions of the mPFC during the thermal threat task. An optogenetic-mediated silencing approach was used that selectively targeted CaMKII-expressing principal neurons, which constitute a large majority of cells in these brain regions (Anastasiades and Carter, 2021). PL and IL were silenced individually in different cohorts of male mice (Fig. 3*A*). While controls only expressed eYFP, the experimental groups expressed the proton-pump ArchT, which produces robust neuronal silencing in response to red-shifted light (Yizhar et al., 2011; El-Gaby et al., 2016). Laser light was delivered through chronically implanted optical fibers (Fig. 3*B* and Extended Data Fig. 3-1). These manipulations were performed throughout the 10 min of training during the threat task, but not during the subsequent recall test (Fig. 3*C*).

10.1523/ENEURO.0140-23.2024.f3-1 Figure 3-1 Reconstruction of the optical fiber placements and the sites of viral infusions for the optogenetic experiments in Figure 3. ***A,*** Mid-sagittal drawing of the mouse brain illustrating the PL and IL regions of the mPFC. ***B-C,*** Coronal photomicrographs of the mPFC. Tissue landmarks and key cytoarchitectonic features of the mPFC (e.g., transitions in the cortical layers) were carefully examined in each mouse to separate the PL and IL regions, and to determine whether there was appropriate bilateral targeting of the individual brain regions. Mice were excluded if the individual targets were missed with the optical fibers or if there was viral leakage. ***D-E,*** Summary of the optical fiber placements (represented with colored tubes) and viral infusion centroids (represented with colored “×” symbols). Numbers within the coronal drawings indicate the anterior-posterior coordinates in millimeters relative to bregma. [eYFP: control groups, ArchT: neural inhibition groups, cc: corpus callosum]. Download Figure 3-1, TIF file.

10.1523/ENEURO.0140-23.2024.f3-2 Figure 3-2 PL and IL inhibition did not affect other behavioral measurements during the thermal tasks. ***A-D,*** During the threat task, PL inhibition did not affect the rate of entries into the threat zone, head dipping into the threat zone, or general locomotion within the entire test apparatus (eYFP: N = 6, ArchT: N = 7). ***E-H,*** During the threat task, IL inhibition did not affect any of these behavioral measurements either (eYFP: N = 8, ArchT: N = 5). ***I-L,*** During the safety task, PL inhibition did not produce any significant effects on these behavioral measurements (eYFP: N = 6, ArchT: N = 7). ***M-P,*** During the safety task, IL inhibition did not affect these behavioral measurements either (eYFP: N = 9, ArchT: N = 6). [*P*-values represent Bonferroni post hoc tests]. Download Figure 3-2, TIF file.

Photoinhibition of either PL or IL produced no significant effects during the thermal threat task. The PL-eYFP and PL-ArchT groups exhibited equivalent levels of avoidance behavior to the thermal threat zone during both sessions (Fig. 3*D*; Group, *F*_(1,11) _= 0.0005, *p *= 0.98; Session, *F*_(1,11) _= 0.54, *p *= 0.48; Interaction, *F*_(1,11) _= 1.57, *p *= 0.24). The IL-eYFP and IL-ArchT groups also exhibited similar levels of avoidance behavior during both sessions (Fig. 3*E*; Group, *F*_(1,11) _= 0.005, *p *= 0.51; Session, *F*_(1,11) _= 0.53, *p *= 0.48; Interaction, *F*_(1,11) _= 0.47, *p *= 0.51). Additional measurements during the threat task also revealed no significant alterations by the PL or IL treatments (Extended Data Fig. 3-2*A* through 3-2H). Thus, learning about the thermal threat zone did not require PL or IL activity.

### Memory formation during thermal safety learning required prefrontal activity

We next examined potential contributions of PL and IL during the thermal safety task. New cohorts of male mice were prepared with either eYFP or ArchT, and optical fibers were chronically implanted to target PL or IL (Fig. 3*A*,*B*; Extended Data Fig. 3-1). Photoinhibition was performed throughout the 10 min of training during the safety task, but not during the subsequent recall test (Fig. 3*F*).

In these experiments, while PL inhibition produced no significant effects, IL inhibition produced an overall impairment in the formation of safety memory. The PL-eYFP and PL-ArchT groups exhibited similar levels of approach behavior to the thermal safety zone during both sessions (Fig. 3*G*; Group, *F*_(1,11) _= 0.06, *p *= 0.81; Session, *F*_(1,11) _= 0.89, *p *= 0.37; Interaction, *F*_(1,11) _= 0.12, *p *= 0.74). In contrast, while the IL-eYFP group exhibited normal performance and recall, the IL-ArchT group exhibited a large reduction in approach behavior during the long-term recall test, despite exhibiting normal performance during training (Fig. 3*H*; Group, *F*_(1,13) _= 5.90, *p *= 0.030; Session, *F*_(1,13) _= 2.96, *p *= 0.11; Interaction, *F*_(1,13) _= 16.2, *p *= 0.0014; post hoc test during the recall test, *p *= 0.0003). No other significant effects were detected with photoinhibition during the safety task (Extended Data Fig. 3-2*I* through 3-2*P*). Therefore, memory formation for the thermal safety zone required neural activity in IL, but not PL.

### Memory formation during thermal threat learning was insensitive to stress

**Figure 4.**
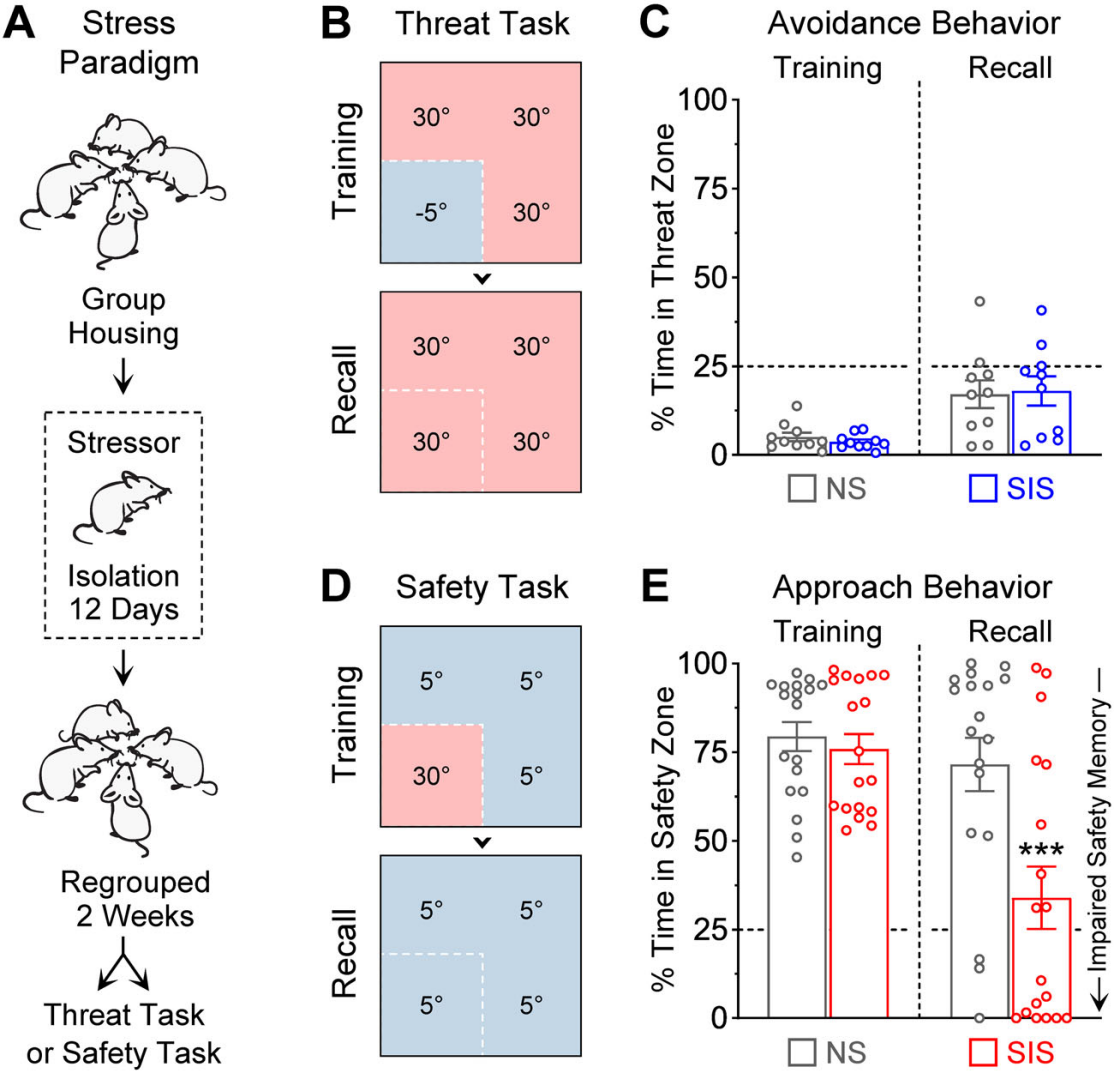
Stress pre-exposure selectively impaired the formation of safety memory. ***A***, A prolonged social isolation stress paradigm was performed in multiple cohorts of mice. While the no-stress control groups (“NS”) always remained in group-housing conditions, the social isolation stress groups (“SIS”) underwent single-housing conditions (i.e., isolation) for 12 consecutive days. After this isolation period, the stress groups were regrouped with their former cagemates for 2 weeks prior to undergoing behavioral testing. Separate cohorts underwent either the thermal threat task or the thermal safety task. ***B***, ***C***, During the threat task, the stress group exhibited normal performance during training and recall of the threat zone during the subsequent test session, compared with controls (NS, *N* = 10; SIS, *N* = 10; Training, *p *> 0.99; Recall, *p *> 0.99). ***D***, ***E***, During the safety task, while the stress group exhibited normal performance during training, they exhibited a robust impairment in the recall of the safety zone the next day, indicating impaired formation of the safety memory (NS, *N* = 18; SIS, *N* = 18; Training, *p *> 0.99; Recall, ***p = 0.0002). See Extended Data Figure 4-1 for additional analyses on stress-induced alterations in bodyweight and Extended Data Figure 4-2 for an additional experiment that compared stress-induced vulnerability in males versus females.

It is well documented that prolonged stress exposure in rodents produces disease-like states that are characterized by significant alterations in threat learning (Conrad et al., 1999; Rau and Fanselow, 2009; Corley et al., 2012). We therefore examined how stress affects memory formation in male mice during the thermal threat task. A social isolation stress paradigm was implemented in which the no-stress controls always remained in group-housing conditions (four mice per cage), whereas mice in the stress groups underwent single-housing conditions (i.e., social isolation) for 12 d (Fig. 4*A*). This period of social isolation was sufficient to produce significant impairments in bodyweight gain, which is typically a good indicator for stressor effectiveness (Extended Data Fig. 4-1*A* and 4-1*B*). After the 12 d of isolation, mice were regrouped with their former cagemates and underwent behavioral testing 2 weeks later in the thermal threat task (Fig. 4*B*). Contrary to other threat learning tasks, no significant effects of stress were observed during the thermal threat task (Fig. 4*C*; Group, *F*_(1,18) _= 0.005, *p *= 0.94; Session, *F*_(1,18) _= 20.0, *p *= 0.0003; Interaction, *F*_(1,18) _= 0.15, *p *= 0.70). Thus, memory formation for the thermal threat zone was insensitive to social isolation stress.

10.1523/ENEURO.0140-23.2024.f4-1 Figure 4-1 Stress-induced alterations in bodyweight. ***A,*** Social isolation stress was performed for twelve days, and bodyweights were measured every other day. ***B,*** Compared to no-stress controls (NS = 18), mice that underwent social isolation stress (SIS = 18) showed a significant impairment in weight gain (Group, *F*_(1,34)_ = 7.57, *P* = 0.009; Time, *F*_(6,204)_ = 7.11, *P* < 0.0001; Interaction, *F*_(6,204)_ = 5.13, *P* < 0.0001; **P < 0.01, ***P < 0.001). ***C,*** Stressed mice were separated into two sub-groups (“Stress Resilient” vs “Stress Susceptible”) based on whether they showed or did not show deficits during the thermal safety task. A cutoff was arbitrarily set at 50%, based on time spent in the safety zone during the recall test. ***D,*** No significant differences were detected in bodyweight between the resilient and susceptible groups (Unpaired T-test: *P* = 0.87). Download Figure 4-1, TIF file.

10.1523/ENEURO.0140-23.2024.f4-2 Figure 4-2 Evaluation of potential sex differences in stress vulnerability during the thermal safety task. ***A,*** Social isolation stress was performed for twelve days in new cohorts of male and female mice. Male-NoStress (M-NS, N = 8), Male-Stress (M-SIS, N = 8), Female-NoStress (F-NS, N = 8), and Female-Stress (F-SIS, N = 8). ***B,*** Changes in bodyweight across the stress days compared to controls (Males: Group, *F*_(1,14)_ = 2.29, *P* = 0.15; Time, *F*_(6,84)_ = 5.86, *P* < 0.0001; Interaction, *F*_(6,84)_ = 1.24, *P* = 0.30) (Females: Group, *F*_(1,14)_ = 0.06, *P* = 0.81; Time, *F*_(6,84)_ = 8.52, *P* < 0.0001; Interaction, *F*_(6,84)_ = 1.39, *P* = 0.23). ***C,*** Behavioral testing in the thermal safety task was conducted twelve days after the cessation of stress. ***D,*** Approach behavior assessed as the time spent within the safety zone (Group, *F*_(3,28)_ = 8.04, *P* = 0.0005; Time, *F*_(1,28)_ = 34.4, *P* < 0.0001; Interaction, *F*_(3,28)_ = 10.7, *P* < 0.0001). Overall, females exhibited more time within the safety zone than males during the training session (all males versus all females, **P = 0.0026). Such sex difference disappeared during the long-term recall test (all males versus all females, *P* > 0.99). Yet, the stressed groups in both males and females exhibited significantly less time within the zone that predicted safety during the recall test (**P = 0.009, ****P < 0.0001), suggesting that stress impaired safety memory formation in a sex-independent manner. Download Figure 4-2, TIF file.

### Memory formation during thermal safety learning was highly sensitive to stress

We then examined the effects of social isolation stress during the thermal safety task. For this, a new cohort of male mice underwent social isolation for 12 d (Fig. 4*A*) and underwent testing in the thermal safety task 2 weeks later (Fig. 4*D*). Despite normal performance during the initial training session, compared with controls the stress group exhibited significantly lower approach behavior to the safety zone during the long-term test (Fig. 4*E*; Group, *F*_(1,34) _= 11.2, *p *= 0.002; Session, *F*_(1,34) _= 13.2, *p *= 0.0009; Interaction, *F*_(1,34) _= 6.18, *p *= 0.018; post hoc test during recall, *p *= 0.0002). While some mice exhibited stress resilience, individual differences were not related to variance in bodyweight loss during stress (Extended Data Fig. 4-1*C* and 4-1*D*). Despite this, the overall large reduction in approach behavior during recall suggests that stress impaired memory formation for the thermal safety zone.

Since previous studies have reported sex differences in safety learning (Krueger and Sangha, 2021), as well as in stress susceptibility (Shansky, 2015; Merz and Wolf, 2017), we then compared the effects of social isolation stress during the thermal safety task in new cohorts of male and female mice (Extended Data Fig. 4-2). Overall, compared with males, females exhibited significantly higher levels of approach to the safety zone during the training session (Bonferroni’s-corrected unpaired *t* test: all males vs all females, *p *= 0.0026). However, such sex difference disappeared during the recall test (Bonferroni’s-corrected unpaired *t* test: all males vs all females, *p *> 0.99). Furthermore, compared with the control groups, the stressed groups exhibited a robust reduction in approach behavior during the recall test in both males (*p *= 0.009) and females (*p *< 0.0001). Therefore, despite robust performance during training, stress altered memory formation for the thermal safety zone in a sex-independent manner.

### Stress uncovered bidirectional influence of IL and PL during the thermal safety task

**Figure 5.**
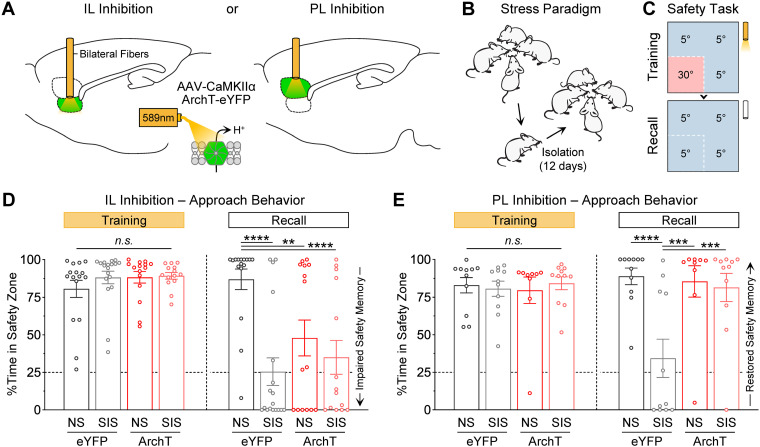
IL inhibition mimicked the effects of stress in unstressed mice, whereas PL inhibition restored the formation of safety memory in stress-exposed mice. ***A***, Optogenetic inhibition strategy. ***B***, Social isolation stress paradigm. ***C***, Photoinhibition was performed during the training session of the thermal safety task. ***D***, IL inhibition produced an impairment in the formation of safety memory that was similar to the deficits produced by the prior pre-exposure to stress (NS-eYFP, *N* = 16; SIS-eYFP, *N* = 18; NS-ArchT, *N* = 15; SIS-ArchT, *N* = 14; **p < 0.01, ****p < 0.0001). ***E***, While PL inhibition did not affect safety learning in no-stress controls, PL inhibition fully restored the ability of stress-exposed mice to form a robust memory for the safety zone, thereby allowing animals to exhibit normal levels of approach behavior during the recall test (NS-eYFP, *N* = 11; SIS-eYFP, *N* = 11; NS-ArchT, *N* = 9; SIS-ArchT, *N* = 11; ***p < 0.001, ****p < 0.0001). See Extended Data Figures 5-1, 5-2, and 5-3 for full histological reconstruction and for additional analyses on individual differences.

Intriguingly, the effect produced by the stress treatment during the thermal safety task was remarkably similar to the effect observed with IL photoinhibition. This made us wonder whether these effects were associated. Thus, we conducted an additional series of experiments that combined the stress pre-exposure and optogenetic photoinhibition treatments. For these, new cohorts of male mice were infused with the ArchT or eYFP viruses in either the IL or PL region (Fig. 5*A* and Extended Data Fig. 5-1). Then, while some groups always remained under group-housing conditions (i.e., no-stress controls, “NS”), other groups underwent social isolation for 12 d (i.e., stressed groups, “SIS”; Fig. 5*B*). Two weeks later, all groups underwent behavioral testing in the thermal safety task, with laser treatments for photoinhibition occurring during the training session, but not during the subsequent recall test (Fig. 5*C*).

10.1523/ENEURO.0140-23.2024.f5-1 Figure 5-1 Fiber placements and viral infusion sites for the optogenetic experiments that also included stress treatments in Figure 5. ***A,*** Summary of IL targeting. ***B,*** Summary of PL targeting. The colored tubes represent the optical fibers, while the “×” symbols represent the viral infusion sites. [NS: no stress, SIS: social isolation stress, eYFP: control fluorophore, ArchT: neural inhibition opsin]. Download Figure 5-1, TIF file.

10.1523/ENEURO.0140-23.2024.f5-2 Figure 5-2 Individual variability in the effects of IL inhibition during the thermal safety task was unrelated to variability in the position of optical fibers or location of viral infusions. ***A,*** Schematic of the safety task with IL inhibition occurring during the training session. ***B,*** Combined IL inhibition data from Fig 3H (ArchT group) and Fig 5D (NS-ArchT group). Mice were bimodally distributed, with some exhibiting robust impairment in safety memory (12/21, 57%), and others exhibiting no impairment (9/21, 43%). ***C,*** Coronal drawings illustrate the position of optical fibers and viral infusion sites in IL. No major differences were appreciated between these two sub-groups. IL targeting seemed similarly distributed across the anterior-posterior, medial-lateral, and dorsal-ventral planes in the two groups. Download Figure 5-2, TIF file.

10.1523/ENEURO.0140-23.2024.f5-3 Figure 5-3 Social status did not contribute to individual variability produced by either stress or infralimbic inhibition during the thermal safety task. ***A,*** Within each homecage, mice were assigned social ranks based on differences in bodyweight, measured prior to any treatment or behavioral testing. Digits within the bars indicate the number of mice per rank (One-way ANOVA: *F*_(3,43)_ = 21.3, *P* < 0.0001; Bonferroni Tests: *P = 0.013, ***P = 0.0009, ****P < 0.0001). ***B-D,*** Mice were further separated into sub-groups based on whether they showed or did not show impairment in safety memory during the safety task. ***B,*** In mice that received the SIS-eYFP treatment (i.e., stress but no photoinhibition) social ranks seemed similarly distributed between the no-impairment and impairment groups. ***C,*** In mice that received the NS-ArchT treatment (i.e., IL photoinhibition but no stress), social ranks also seemed similarly distributed between the two groups. ***D,*** Finally, in mice that received the SIS-ArchT double treatment (i.e., stress and IL photoinhibition), social ranks also seemed similarly distributed between the no-impairment and impairment groups. ***E,*** Combined distributions, based on social ranks and deficits in the safety task. No significant difference was detected based on a chi-square test. Download Figure 5-3, TIF file.

In the IL experiment, while the stress and ArchT treatments did not affect the emergence of approach behavior during the training session, these treatments produced similar deficits in the subsequent recall of safety memory (Fig. 5*D*; Group, *F*_(3,59) _= 4.93, *p *= 0.004; Session, *F*_(1,59) _= 48.9, *p *< 0.0001; Interaction, *F*_(3,59) _= 8.48, *p *< 0.0001). Post hoc comparisons confirmed that compared with the double-control group, the stress-exposed group that did not receive IL inhibition exhibited significantly lower approach behavior during recall (NS-eYFP vs SIS-eYFP; *p *< 0.0001). Similarly, the no-stress controls that only received IL inhibition also exhibited significantly lower approach behavior during recall (NS-eYFP vs NS-ArchT; *p *= 0.0027). Furthermore, the double-treatment group that received both stress and IL inhibition also exhibited significantly lower approach behavior during recall (NS-eYFP vs SIS-ArchT; *p *< 0.0001). These results suggest that stress produced deficits in the formation of safety memory through reductions in neuronal activity in IL. This idea is consistent with previous studies reporting that prolonged stressors, including social isolation, significantly reduce neuronal activity in IL (Lee et al., 2011; Park et al., 2021).

Thus far, it was clearly evident that not all the subjects that received IL inhibition exhibited deficits in the formation of safety memory. One might think that such discrepancy could have been attributed to the possibility that distinct portions of IL (e.g., anterior vs posterior) may have been more involved in safety memory than others. To examine this, we evaluated variability in the position of optical fibers and the sites of viral vector injections within IL and made comparisons between mice that exhibited impairment in safety memory versus mice that did not exhibit impairment. Both groups showed similar distributions of optical fiber placement and viral injection sites across the AP, ML, and DV planes (Extended Data Fig. 5-2). Another factor that could have potentially contributed to individual differences in safety memory (*produced by either IL inhibition or stress exposure*) was differences in social status. That is, higher social ranks could have conferred resilience to IL inhibition or to stress exposure (Zhou et al., 2017; Larrieu and Sandi, 2018). To examine this, we used baseline bodyweight prior to any treatment as an indicator of social status, since dominant mice tend to be larger than subordinates (Fulenwider et al., 2022). This analysis revealed that all social ranks were similarly distributed between the subgroups that exhibited or did not exhibit impairment in safety memory (Extended Data Fig. 5-3). Therefore, such individual differences in safety memory may have been attributed to other unforeseen factors that were not measured in this study (e.g., hormonal adaptations or compensations by other brain regions).

In contrast to IL, PL inhibition produced a surprising rescue effect in the ability of stress-exposed mice to establish robust long-term memory for the safety zone (Fig. 5*E*; Group, *F*_(3,38) _= 6.21, *p *= 0.0015; Session, *F*_(1,38) _= 2.45, *p *= 0.31; Interaction, *F*_(3,38) _= 4.49, *p *= 0.0086). Replicating some of the findings above, the stress group that did not receive PL inhibition exhibited significantly lower approach behavior during recall (NS-eYFP vs SIS-eYFP; *p *< 0.0001), whereas the no-stress group that received PL inhibition exhibited normal levels of approach behavior during recall (SIS-eYFP vs NS-ArchT; *p *= 0.0002). However, the stress group that received PL inhibition also exhibited high levels of approach behavior during recall (SIS-eYFP vs SIS-ArchT; *p *= 0.0004). Therefore, PL inhibition fully restored memory formation during the safety task in stress-exposed mice. These findings are consistent with the idea that deficits in the formation of safety memory after stress could be attributed to elevated PL activity. This idea is supported by previous studies reporting stress-induced elevations in PL activity (Lee et al., 2011). Together with the IL findings above, it appears that have bidirectional influence over the formation of safety memory during thermal threat, with IL activity normally facilitating this function, while PL activity dampens this function especially after stress-induced alterations.

### The IL and PL manipulations did not affect general anxiety, locomotion, or place preference

**Figure 6.**
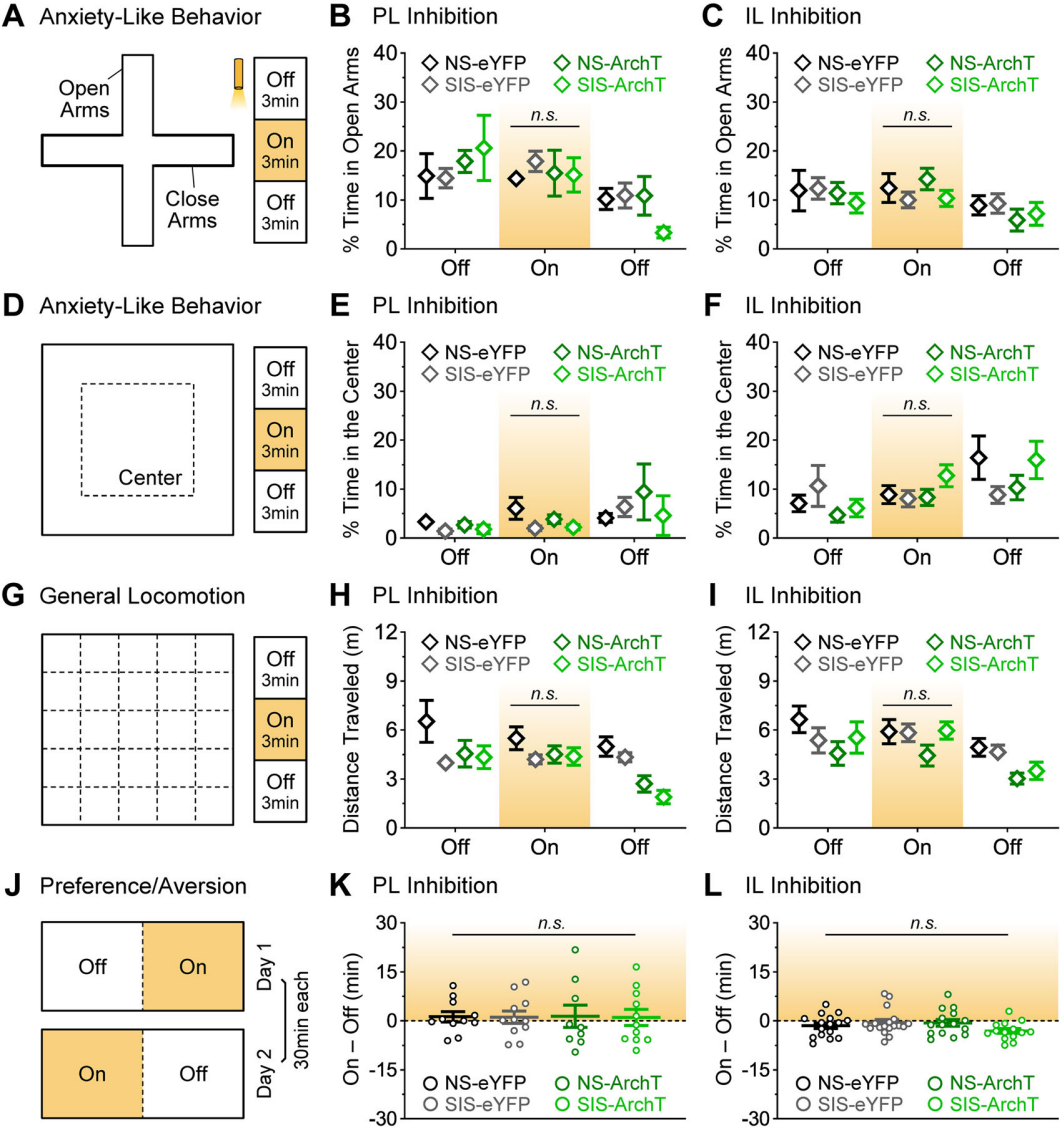
Other behaviors related to general anxiety, locomotion, and place preference were not affected by the photoinhibition or stress treatments. ***A–C***, Anxiety-like behavior in the elevated-plus maze test, assessed as the average time spent in the open arms during each 3 min laser epoch (PL, all *p's* > 0.99; IL, all *p's* > 0.75). ***D–F***, Anxiety-like behavior in the open-field test, assessed as the average time spent in the center of the arena during each 3 min laser epoch (PL, all *p's* > 0.55; IL, all *p's* > 0.65). ***G–I***, Locomotor activity in the open-field test, assessed as the total distance traveled in the arena during each 3 min laser epoch (PL, all *p's* > 0.63; IL, all *p's* > 0.47). ***J–L***, Real-time place preference test, analyzed as the total time spent in the Laser-On minus Laser-Off sides (PL, all *p's* > 0.99; IL, all *p's* > 0.28). Same animals as in Figure 5. PL-NS-eYFP, *N* = 11; PL-SIS-eYFP, *N* = 11; PL-NS-ArchT, *N* = 9; PL-SIS-ArchT, *N* = 11; IL-NS-eYFP, *N* = 16; IL-SIS-eYFP, *N* = 18; IL-NS-ArchT, *N* = 15; IL-SIS-ArchT, *N* = 14; *n.s.*, not significant.

Prefrontal cortical regions regulate anxiety-related behaviors (Adhikari et al., 2011; Felix-Ortiz et al., 2016). This raises the question of whether the effects observed during the thermal safety task could be attributed to alterations in general anxiety. To rule out this possibility, we conducted additional behavioral assays. During an elevated-plus maze test (Fig. 6*A*), anxiety levels were evaluated as the percentage of time that mice spent in the open arms of the maze. No significant differences on anxiety were detected across groups with PL photoinhibition (Fig. 6*B*; two-way ANOVA: Group, *F*_(3,38) _= 0.15, *p *= 0.93; Laser Epoch, *F*_(2,76) _= 7.32, *p *= 0.0021; Interaction, *F*_(6,76) _= 1.09, *p *= 0.38; post hoc tests for the Laser-On epoch, all *p's *> 0.99). No significant differences on anxiety were detected across groups with IL photoinhibition either (Fig. 6*C*; two-way ANOVA: Group, *F*_(3,59) _= 0.22, *p *= 0.89; Laser Epoch, *F*_(2,118) _= 5.94, *p *= 0.0048; Interaction, *F*_(6,118) _= 0.39, *p *= 0.47; post hoc tests for the Laser-On epoch, all *p's *> 0.75). During an open-field test (Fig. 6*D*), anxiety levels were evaluated as the percentage of time that mice spent in the center zone of the arena. No significant differences on anxiety were detected across groups with PL photoinhibition (Fig. 6*E*; two-way ANOVA: Group, *F*_(3,38) _= 0.70, *p *= 0.56; Laser Epoch, *F*_(2,76) _= 3.45, *p *= 0.064; Interaction, *F*_(6,76) _= 0.71, *p *= 0.65; post hoc tests for the Laser-On epoch, all *p's *> 0.55). No significant differences on anxiety were detected across groups with IL photoinhibition either (Fig. 6*F*; two-way ANOVA: Group, *F*_(3,59) _= 0.79, *p *= 0.51; Laser Epoch, *F*_(2,118) _= 6.08, *p *= 0.0044; Interaction, *F*_(6,118) _= 1.71, *p *= 0.13; post hoc tests for the Laser-On epoch, all *p's *> 0.65). Thus, the photoinhibition treatments in this study were insufficient to affect anxiety, and thereby this possibility was ruled out to explain the effects on safety learning.

Possible effects in general locomotion could be another factor that may have confounded results during the thermal safety task. To further rule out this possibility, we evaluated general locomotor activity during the open-field test as the cumulative distance that mice traveled within the arena (Fig. 6*G*). No significant differences were detected on distance traveled across the groups with PL manipulations (Fig. 6*H*; two-way ANOVA: Group, *F*_(3,38) _= 4.17, *p *= 0.012; Laser Epoch, *F*_(2,76) _= 7.62, *p *= 0.0033; Interaction, *F*_(6,76) _= 1.92, *p *= 0.089; post hoc tests for the Laser-On epoch, all *p's *> 0.63). No significant differences on distance traveled were detected across the groups with IL manipulations (Fig. 6*I*; two-way ANOVA: Group, *F*_(3,59) _= 1.98, *p *= 0.13; Laser Epoch, *F*_(2,118) _= 14.9, *p *< 0.0001; Interaction, *F*_(6,118) _= 0.89, *p *= 0.50; post hoc tests for the Laser-On epoch, all *p's *> 0.47). Thus, the possibility of confounding effects through altered locomotor activity was also ruled out.

Finally, another factor that could have confounded results during the thermal safety task was that prolonged optogenetic manipulations on their own could produce rewarding-like or aversive-like effects capable to promote place preference or aversion. A real-time place preference assay was conducted to evaluate this possibility, in which the PL or IL photoinhibition treatments were performed in a closed-loop manner when mice entered into one half of a rectangular-shaped arena, but not when entering into the other half (Fig. 6*J*). No signs of place preference or aversion were detected with either PL inhibition (Fig. 6*K*; one-way ANOVA: *F*_(3,38) _= 0.005, *p *> 0.99) or IL inhibition (Fig. 6*L*; one-way ANOVA: *F*_(3,59) _= 1.68, *p *= 0.18). Therefore, the possibility of nonspecific effects through alterations in place preference or place aversion could also be ruled out to explain the effects observed during the thermal safety task.

## Discussion

In this study, we designed a novel seminaturalistic paradigm to evaluate threat and safety learning when environments involved spatially distributed thermal reinforcers. We focused on the IL and PL subregions of the mPFC to provide new insights into mechanisms supporting these learning processes. We observed that neural activity in neither of these regions was required for learning about a thermally threatening zone. However, both regions played critical roles for regulating memory formation for a thermally safe zone. While IL activity promoted robust safety memory during normal conditions, PL activity suppressed safety memory especially after mice underwent stress. In addition, while selective inhibition of IL mimicked the detrimental effects of stress on safety memory, selective inhibition of PL was sufficient to restore the ability of stressed mice to establish robust safety memory. These findings highlight the importance of mPFC processing for bidirectional regulation of safety learning when naturalistic conditions involve spatio-thermal contingencies and suggest that stress produces deficits in safety learning that are mediated through alterations in the IL and PL regions.

### Advantages and potential disadvantages of the new thermal tasks

Despite their simplicity, the spatial-based learning tasks described here represent new opportunities to evaluate threat and safety learning from a perspective that is more ethologically relevant. Notably, mice also exhibited rapid learning of the spatio-thermal contingencies that predicted threat versus safety, and the memory representations that resulted from these learning processes were easily recalled later on without the requirement of further reinforcement. Another valuable aspect of the new thermal tasks is that threat and safety learning were associated with distinctive yet related adaptive behaviors. While threat learning was driven by avoidance behavior, thermal safety learning was driven by approach behavior. In contrast, during other traditional paradigms that involve cue or contextual discrimination for predictions or omissions of electric shock, while threat learning is typically associated with conditioned freezing responses, safety learning is rather inferred from the lack or reduction in freezing (Rescorla, 1969; Sangha et al., 2013; Sosa and Ramírez, 2019). Other advantages of the thermal tasks have to do with limited spreading of the naturalistic reinforcers, which in the present study were confined to particular spatial zones within the same controlled environment. In contrast, in other paradigms involving reinforcers such as predator odors, food aromas, or neutral scents (Blanchard et al., 2005; Takahashi et al., 2008), the odorants tend to spread throughout the test arena and beyond, which could easily confound separations and interpretations for threat and safety learning.

In addition to be fast, ethologically relevant, and easy to perform, the present thermal tasks could also facilitate evaluations in female subjects. A growing body of preclinical literature suggests that females have greater vulnerability for fear generalization and dysregulation during threat, effects that are thought to be attributed to deficits in the mechanisms associated with safety learning. For example, profound deficits have been reported in female rodents during fear extinction, cue discrimination, and conditioned inhibition procedures (Fenton et al., 2014; Day et al., 2016; Greiner et al., 2019; Krueger and Sangha, 2021). Yet, these traditional procedures have also yielded numerous mixed results in females (Milad et al., 2009; Foilb et al., 2018; Clark et al., 2019; Velasco et al., 2019; Trask et al., 2020). In contrast, our thermal safety task proved to be highly effective in female mice to produce robust safety learning. For instance, the majority of the tested females (15/16, 93.8%) showed robust approach to the thermal safety zone during the training session (Extended Data Fig. 4-2*D*). In addition, most of the females that did not receive stress (7/8, 87.5%) exhibited robust long-term recall of the safety memory, whereas most of the females that underwent stress (7/8, 87.5%) exhibited significant impairment during recall. Such striking results are very tempting for future studies to implement the thermal task in females to facilitate evaluations for safety learning.

A possible disadvantage of the thermal tasks is that they do not include a trial-based structure. This could introduce multiple challenges when trying to evaluate the neural correlates associated with these types of learning. For instance, evaluations of neural activity with in vivo electrophysiology, calcium imaging, and other complementary techniques often require trial-based structures to sample sufficient data for better statistical power. But the lack of a trial-based structure in the thermal tasks, as implemented in this study, could result in issues of undersampling due to reduced number of instances in which animals explore the threat zone or enter and exit the safety zone (e.g., our mice crosses the thermal zones approximately 10 times during the 10 min of training and no more than three times during the 3 min of the recall test). To overcome this challenge, future studies could implement modifications to the thermal tasks, such as extending the length of the sessions, perform multiple daily training sessions, or introduce a trial-based design in which mice are removed and reintroduced into the arena multiple times during any given daily session, similar to other spatial learning tasks (Morris, 1984; Vorhees and Williams, 2006). Another strategy could be to occasionally drop food pellets in different parts of the arena to persuade animals to either enter the threat zone or leave the safety zone more often to gather food. However, such strategy would be expected to create even more complex scenarios with competing naturalistic motivational drives (i.e., threat avoidance vs food collection, safety approach vs food foraging, etc.). Such scenarios could serve as novel avenues for new research projects that focus on the competition of multidimensional naturalistic behaviors (Amir et al., 2015; Bravo-Rivera and Sotres-Bayon, 2020; Fernandez-Leon et al., 2021; Wang et al., 2021).

### Lack of prefrontal involvement during the thermal threat task

Our initial prediction was that PL and IL could play essential roles during the thermal threat task. This prediction was based on compelling evidence that both PL and IL exhibit prominent neuronal signals that are essential for the learning and selection of defensive behavior during situations involving threat. Such observations have been made during a variety of behavioral paradigms, including cue–shock conditioning (Gilmartin and McEchron, 2005; Burgos-Robles et al., 2007, 2009; Santini et al., 2008; Giustino et al., 2016; Bloodgood et al., 2018), context–shock conditioning (Rozeske et al., 2015; Corches et al., 2019), shock avoidance (Bravo-Rivera et al., 2014; Diehl et al., 2018; Jercog et al., 2021; Martin-Fernandez et al., 2023), and several other paradigms that involve conflicts between threat and reward (Burgos-Robles et al., 2017; Capuzzo and Floresco, 2020; Fernandez-Leon et al., 2021; McLaughlin et al., 2021). However, during our thermal threat task, PL and IL inhibition failed to produce any effect.

A possible explanation for the lack of effects during the threat task is that the thermal reinforcer (−5°C) perhaps mostly triggered undeliberate fixed reactions (i.e., stimulus–response reflexes, e.g., paw withdrawal or flinch due to pain; Foster et al., 2015; LeDoux and Daw, 2018) that bypassed the mPFC. Nonetheless, mice consistently exhibited learning curves during training in which avoidance behavior emerged as mice experienced the cold stimulus. Furthermore, during the subsequent recall test, mice continued to exhibit avoidance to the correct spatial zone even when the cold stimulus was no longer present. These observations strongly suggest that mice performed avoidance in a calculated manner. In addition, the mPFC has been identified as a key brain region to evaluate whether thermal stimuli are noxious or pleasant (Egan et al., 2005; Aizawa et al., 2019). Therefore, according to theories for the evaluation of thermal stimuli, avoidance learning, and executive control of behavioral responses during threat, we should have seen some mPFC involvement during the thermal threat task.

Conversely, the lack of mPFC involvement was perhaps due to differences between passive and active avoidance. During the threat task, avoidance behavior was mostly performed in a passive manner. That is, despite some experience with the cold stimulus, mice mostly withheld from entering into the cold zone to prevent harm. While active avoidance requires the mPFC (Martinez et al., 2013; Moscarello and LeDoux, 2013; Bravo-Rivera et al., 2014; Diehl et al., 2019, 2020), some evidence suggests that passive avoidance does not depend on mPFC (Brennan et al., 1977; Capuzzo and Floresco, 2020). Future studies could implement new variations of the thermal threat task to disentangle mPFC involvement during different forms of avoidance to cold stimuli.

### Prefrontal contributions during the thermal safety task and beyond

IL activity has been traditionally viewed as a fundamental mechanism for modulating threat-related fear responses. This view is supported by numerous observations that prominent excitatory signals, as well as a variety of other physiological and molecular events, occur in IL during the efficient reduction of fear responses when cues that used to predict threat no longer make such prediction (i.e., fear extinction; Milad and Quirk, 2002; Burgos-Robles et al., 2007; Quirk and Mueller, 2008; Santini et al., 2008; Bloodgood et al., 2018). Furthermore, manipulations that either increase or decrease neuronal activity in IL have been shown to produce significant enhancement or impairment, respectively, in fear extinction (Vidal-Gonzalez et al., 2006; Sierra-Mercado et al., 2011; Fucich et al., 2018). Yet, the view of IL as an essential modulator of fear responses during extinction was further expanded into the notion that IL activity represents overall states of safety. This view is supported by additional observations in which IL activity was necessary to prevent inappropriate fear responding during the presentation of neutral cues or during the presentation of cues that were trained to explicitly omit punishment (Sangha et al., 2014; Kreutzmann et al., 2020; Ng and Sangha, 2022). In addition, IL activity has also been correlated with contextual safety and contextual-dependent inhibition of fear responses (Thompson et al., 2010; Corches et al., 2019; Brockway et al., 2023; Shan et al., 2023). The present study furthers the role of IL for establishing representations of spatially distributed thermal safety. That is, when mice were challenged within a thermally challenging environment, mice needed IL activity to memorize the precise location in which thermal safety could be found. Altogether, these observations are consistent with the notion that IL activity facilitates representations of overall safety, regardless of whether safety is predicted by specific cues, contexts, of precise locations within environments containing threat or the possibility of threat.

In contrast to IL, our findings suggest that PL activity is detrimental for safety memory. At first, our PL inhibition treatment during normal conditions (i.e., no stress) produced no effects during the safety task, which is consistent with a previous report that PL inactivation did not affect safety signals to suppress threat-related fear (Sangha et al., 2014). However, our subsequent experiment that also involved a stress treatment, which we showed multiple times to produce profound deficits in the formation of safety memory, it was revealed that PL inhibition rescued the ability of stress-exposed mice to form robust safety memory. Together with the observation that IL inhibition mimicked the deficits produced by stress, these findings suggest that IL and PL oppose each other to bidirectionally modulate safety memory, with IL activity promoting this function during normal conditions while PL activity suppresses safety memory during stress-induced perturbations.

### How prefrontal areas may receive spatial and thermal signals to promote safety memory?

Compelling evidence suggests that the mPFC receives spatial and contextual information directly from the hippocampus to promote learning and behavioral adaptation. In particular, the ventral hippocampus (vHPC) provides dense glutamatergic inputs that form heterogeneous connectivity within the mPFC for dynamic regulation of neuronal activity and function. For instance, a recent study revealed that a glutamatergic projection that originates in the superficial layers of CA1 preferentially connects with inhibitory neurons in the mPFC to promote behavior associated with approach, whereas another glutamatergic projection that originates in the deep layers of CA1 preferentially connects with pyramidal neurons in the mPFC to promote behavior associated with avoidance (Sánchez-Bellot et al., 2022). Projections from vHPC to mPFC have also been implicated in the modulation of threat-induced fear expression, fear renewal, fear extinction, and safety signaling (Sotres-Bayon et al., 2012; Moscarello and Maren, 2018; Meyer et al., 2019). Therefore, vHPC inputs to mPFC seem like great candidates to further evaluate the neural circuits contributing to the encoding of spatio-thermal contingencies for safety.

There are several options for how thermal-related information may reach the mPFC to promote learning and behavioral adaptation. One possibility is through thalamic nuclei (e.g., paraventricular nucleus, ventral posterior lateral nucleus, and/or medial nucleus), which are essential for the processing of temperature and pain signals originating in the body (Kenshalo et al., 1980; Kanai et al., 2022; Viena et al., 2022; Leva and Whitmire, 2023). Another option is through the lateral parabrachial nucleus which responds to thermal stimuli, modulates adaptive behaviors such as avoidance during thermal stimulation, and sends direct glutamatergic projections to the mPFC (Grady et al., 2020; Yahiro et al., 2023). Another option is through the ventromedial hypothalamic nucleus, which receives inputs from the lateral parabrachial nucleus, responds to thermal stimuli, and also moderates thermoregulatory behaviors (Norris et al., 2021; Feng et al., 2022). Alternatively, the basolateral amygdala may transform thermal stimuli into valence signals for eventual relay into the mPFC (McGarry and Carter, 2017; O’Neill et al., 2018). While most of these inputs to mPFC still remain untested, their assessment may provide valuable insights into the mechanisms underlying the encoding of thermal safety.

### How stress may have affected prefrontal activity to produce deficits in safety memory?

Chronic, repeated, or prolonged exposure to stress alters cognitive function, produces dysregulation in negative emotions such as fear, and increases risk for pathological states such as anxiety and post-traumatic stress disorder (McEwen and Sapolsky, 1995; McEwen, 2005; Gillespie et al., 2009; Wellman et al., 2018). In preclinical studies, it has been well documented that stress enhances susceptibility for enhanced fear during threat learning and extinction (Conrad et al., 1999; Rau and Fanselow, 2009; Corley et al., 2012; Maren and Holmes, 2016; Åhs et al., 2017; Algamal et al., 2021). Despite this, we observed no effects of stress during the novel thermal threat task. Since alterations in IL and PL have been identified as key mediators of stress-related disease (Izquierdo et al., 2006; Holmes and Wellman, 2009), it is then possible that the lack of effects of stress during the thermal threat task was attributed to the lack of involvement of the IL and PL regions during this task.

In contrast, stress produced robust impairments during the thermal safety task. Such impairments were likely mediated through both IL and PL. Supporting this claim, while IL inhibition mimicked the effects of stress on safety memory, PL inhibition restored this function in stressed mice. These findings suggests that IL and PL responded in opposite manners during stress, with IL likely reducing its activity and PL likely increasing its activity. This interpretation is consistent with previous studies reporting reduced IL activity and elevated PL activity as rodents received repeated or prolonged stress exposure, including social isolation (Lee et al., 2011; Park et al., 2021). However, our findings showing significant effects of stress in safety learning in both male and female mice are not precisely consistent with recent studies reporting mixed effects during a conditioned inhibition task. In one study, male rats received footshock stress prior to the conditioned inhibition task, and no effects were observed in the ability of the safety cue to reduce fear responses to the threat cue (Woon et al., 2020). In another study, male mice exhibited impairment in conditioned inhibition of fear after footshock stress, but female mice did not show an impairment (Adkins et al., 2022). In another study, chronic unpredictable stress impaired conditioned inhibition in adult male mice, but not in adolescent mice (Meyer et al., 2021). Overall, it seems that the conditioned inhibition task has yielded contradicting results on the effects of stress on safety learning, whereas our thermal safety task every time produced robust deficits in safety learning.

### Model of balanced prefrontal activity to promote the formation of safety memory

**Figure 7.**
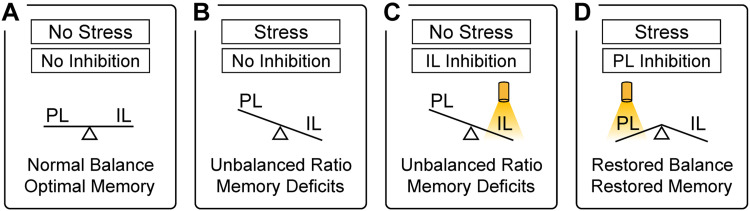
Proposed working model of balanced activity in the prefrontal cortex for the regulation of safety memory formation. ***A***, During normal conditions, balanced activity between PL and IL is suitable for optimal safety memory. ***B***, Stress can produce unbalanced activity between PL and IL, with increased PL and reduced IL (Lee et al., 2011; Park et al., 2021), to produce significant alterations in the formation of safety memory. ***C***, The effects of stress can be mimicked with selective photoinhibition of IL. ***D***, Selective photoinhibition of PL could restore the balance of prefrontal activity, thereby producing a full rescue in the formation of safety memory.

Based on the present findings, we propose a model in which balanced activity between PL and IL promotes the formation of safety memory (Fig. 7*A*). However, stress could tilt the balance between these prefrontal regions, with PL activity getting augmented and IL activity getting reduced, to produce deficits in safety memory (Fig. 7*B*). Furthermore, artificial manipulations that affect the balance between PL and IL activity in healthy controls are sufficient to mimic the effects of stress in the formation of safety memory (Fig. 7*C*). Finally, manipulations capable of restoring the balance between PL and IL activity after stress are sufficient to fully rescue safety memory formation (Fig. 7*D*).

### Implications for fear and stress-related disorders

Consistent with our model of balanced prefrontal activity, it is well documented that unbalanced prefrontal systems characterize various stress-related disorders, including PTSD. For instance, the dorsal anterior cingulate cortex (dACC) in humans, which is a functional homolog to the PL region in rodents, exhibits enhanced resting metabolic activity and potentiated activation during the processing of threat cues in PTSD patients (Milad et al., 2007a; Shin et al., 2009; Marin et al., 2016). In contrast, the ventromedial prefrontal cortex (vmPFC) in humans, which is a functional homolog to the IL region in rodents, exhibits reduced resting metabolic activity and diminished activation during extinction cues in PTSD patients (Milad et al., 2007b; Marin et al., 2016). These prefrontal pathologies are thought to be key mediators of improper signaling of threat even during the presentation of neutral or safety cues to produce fear generalization and profound dysregulation of emotions and behavior (Grillon and Morgan, 1999; Jovanovic et al., 2010, 2012). Yet, how these prefrontal pathologies contribute to specific dysregulation in the formation safety memory in humans remains poorly understood. Observations from the present study encourage further translational efforts to continue disentangling the alterations that occur in the mechanisms governing threat versus safety in the human brain and suggest that potential interventions aimed at restoring the balance within prefrontal cortical systems could serve as powerful strategies to improve safety memory in PTSD and other stress-related disorders.

## Synthesis

Reviewing Editor: Jonathan Lee, University of Birmingham Decisions are customarily a result of the Reviewing Editor and the peer reviewers coming together and discussing their recommendations until a consensus is reached. When revisions are invited, a fact-based synthesis statement explaining their decision and outlining what is needed to prepare a revision will be listed below. The following reviewer(s) agreed to reveal their identity: Ryan Parsons.

The manuscript was reviewed by 2 reviewers and the reviewing editor. We agree that the novel behavioral paradigm is interesting and useful. The experiments have a sound rationale and the results will be of interest to the community. The full reviews are appended below, to which a point-by-point response is requested. We would highlight in particular the themes of:

1. the need to explore further and clarify the nature of the behavior in the novel paradigm. Interpretative claims are made (e.g. threat vs safety learning; that this is learned behavior at all) that are not necessarily substantiated. Suggestions are made for further analysis of the behavior, as well as the potential utility of additional control conditions (to enable certain conclusions to be drawn).

2. The discussion of the use of male mice only needs to be more substantive, and it should be made clearer in the abstract that a single sex was used.

We would also note that the methodological information of the behavioral procedures needs to be more detailed. For example, were the local and distal cues consistent (i.e. the testing box always in the same place and orientation)? Where were the mice placed into the box on each session?

_____________________________

*Reviewer 1*

Overall, I think this is a strong paper and will garner interest from readers interested in aversive learning the cortical control of motivated behavior. My main comments focus on the behavioral task, which is novel and interesting, but that might also require additional data/analyses to convincingly demonstrate that the mice are indeed learning to avoid the cold stimulus vs expressing avoidance behavior towards an innately aversive stimulus that they are able to detect without coming into direct contact with cold stimulus (via corneal cold receptors).

This concern can be addressed by simply examining avoidance behavior minute by minute over the course of the training session. The presence of an acquisition curve where animals come into contact with the cold zone more often early on in the session and then express avoidance later would be expected if the animals are learning avoidance. Steady state avoidance levels throughout the training session would suggest an innate mechanism.

To strengthen the conclusion that animals are learning avoidance based on the presence of the wall cues and not some other cue the animals might pick up on, it would be worthwhile to test the animals in chamber without those cues.

The fact that there are session effects (i.e. mice are showing less avoidance during the recall test) in the threat task is not discussed. This would seem to support an interpretation that either the memory of the learned avoidance is weak or that once the aversive stimulus is removed during recall the animals distribute more time in the place whether the stimulus was located during training?

I am curious as to what the thinking was behind referring to these tasks as 'threat' and 'safety' tasks given that both contain the same stimuli.

Were mice of both sexes used?

The experiments using optogenetics are well done, I especially commend the numerous behavioral controls that help rule out alternative interpretations of the PL/IL inhibition effects on the thermal tasks. The data are also presented in a fashion that make the figures very easy to read.

*Reviewer 2*

This study introduces a novel behavioral task to assess threat avoidance and safety approach based on thermal and spatial stimuli. Consistent with other paradigms of threat/safety, the authors find a critical role of the PL and IL in regulating these behaviors. This seems to indicate the PL and IL have a generalized function in threat and safety given the similarities across tasks, with hyperactivity in PL biasing behavior against safety. The authors combined social isolation stress with PL inhibition to show a return to optimal safety learning, compared to stress without PL inhibition, and to IL inhibition in stress-free mice. The thermal based behaviors seem robust and quick to learn, but below I note that there are very large ranges in % time in the safety zone with IL manipulations that should be scrutinized in greater detail. I also have other suggestions and questions that need addressing that I think would make the manuscript and conclusions stronger.

Since there is a contextual element to the task, what exactly is disrupted in experiments where % time in safety zone is disrupted? The knowledge that there is a safety zone, or the knowledge of where the safety zone is?

Fig 3, There is a very large range in % time in the safety zone, indicating some mice were resilient to the stressor, while others were susceptible and showed poor safety recall. Are there any other measures collected in these mice that could correlate with % time in safety zone? E.g. the more body weight lost, the worse the safety memory?

Figs 2, 4: There are also very large ranges in recall with IL training manipulation. Is it possible that the handful of animals with high % time in safety zone had placements close to the PL/IL border, while those with true safety impairment were located more ventral? If so, this would strengthen the argument that inhibiting the PL, even near the border of the IL, can reverse the stress effect on safety. Perhaps running a correlation of % time in safety zone against the DV estimate of the optic fiber for all animals with IL and PL inhibition. One would expect a nice correlation of more dorsal placements showing more % time in safety, and v.v. This would also get around the difficulty of sometimes being able to see the definitive border between IL and PL.

I cannot see the optical fiber tip in the supplementary fig 2c showing PFC histology.

I did not see a justification of why only males were included in the study, and there was an inadequate discussion of sex differences observed in safety or avoidance studies (there were only a couple of sentences at the very end of the discussion).

The discussion is limited in comparing the thermal safety/threat task to just Pavlovian cued safety/threat tasks, but this is not a fair comparison. This thermal safety/threat approach/avoidance task would be better compared to instrumental tasks, like active or passive avoidance. There are a large number of papers demonstrating a critical role of the PL in these avoidance task that need to be reviewed here and integrated with the presented data. A discussion of PL and IL's roles in controllable stressor is also warranted given that the mice in this task are able to control their exposure to the stressor by either avoiding the threat zone or approaching the safety zone. There are also sex differences in these other tasks (both behaviorally as well as their neuronal correlates) that should be addressed given the authors did not include females in this study.

There should be a discussion regarding how the PL and IL receive thermal sensory information, as well as how these regions receive spatial information since the recall session relies on remembering the spatial cues associated with the threat/safety zones.

Some references that should be included and discussed

1.Meyer et al, showing vHIP input to PL is necessary for conditioned inhibition/safety in both mice and humans.

https://pubmed.ncbi.nlm.nih.gov/31822612/

2.Giustino, Fitzgerald, Maren show neural activity within PL vs IL correlated with differential freezing: high freezing correlated with high spontaneous firing rates in PL in comparison to IL

https://pubmed.ncbi.nlm.nih.gov/27776157/

3.Ng and Sangha show increased IL activity to a conditioned inhibitor that is negatively correlated with freezing behavior.

https://pubmed.ncbi.nlm.nih.gov/36411540/

Visual abstract:

The big X through the entire brain is confusing and I recommend removing. I like the little mouse schematics showing avoidance of threat and more approach to safety. I do not really like the "Safety Learning" table to the right; I think modifying Fig 6 would be a better visual summary.
