# Supplementary figures and images for: Prefrontal Regulation of Safety Learning during Ethologically Relevant Thermal Threat
Source: eNeuro. 2024 Feb 9;11(2):ENEURO.0140-23.2024. doi: 10.1523/ENEURO.0140-23.2024 (PMC10903390; doi:10.1523/ENEURO.0140-23.2024)

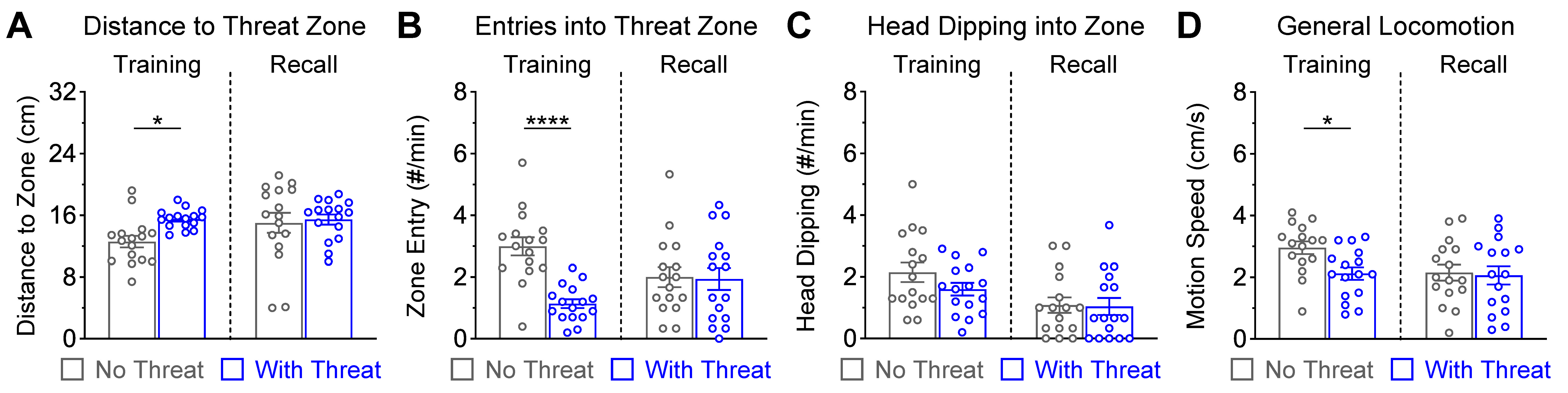

Supplement: Figure 1-1 — Additional measurements during the thermal threat task. A, Distance to the threat zone, measured from the center of the mouse body to the center of the zone (Group, F(1,30) = 2.77, P = 0.11; Time, F(1,30) = 3.62, P = 0.067; Interaction, F(1,30) = 3.88, P = 0.058; Training: *P = 0.034; Recall: P > 0.99). B, Entries into the threat zone (Group, F(1,30) = 8.71, P = 0.006; Time, F(1,30) = 0.15, P = 0.70; Interaction, F(1,30) = 12.6, P = 0.0013; Training: ****P < 0.0001; Recall: P > 0.99). C, Head dipping into the threat zone (Group, F(1,30) = 0.94, P = 0.34; Time, F(1,30) = 13.3, P = 0.001; Interaction, F(1,30) = 1.27, P = 0.27; Training: P = 0.30; Recall: P > 0.99). D, General locomotion in the arena (Group, F(1,30) = 2.54, P = 0.12; Time, F(1,30) = 5.48, P = 0.026; Interaction, F(1,30) = 4.01, P = 0.054; Training: *P = 0.037; Recall: P > 0.99). [No Threat: N = 16, With Threat: N = 16]. Download Figure 1-1, TIF file. [file eneuro-11-ENEURO.0140-23.2024-s005.tif]

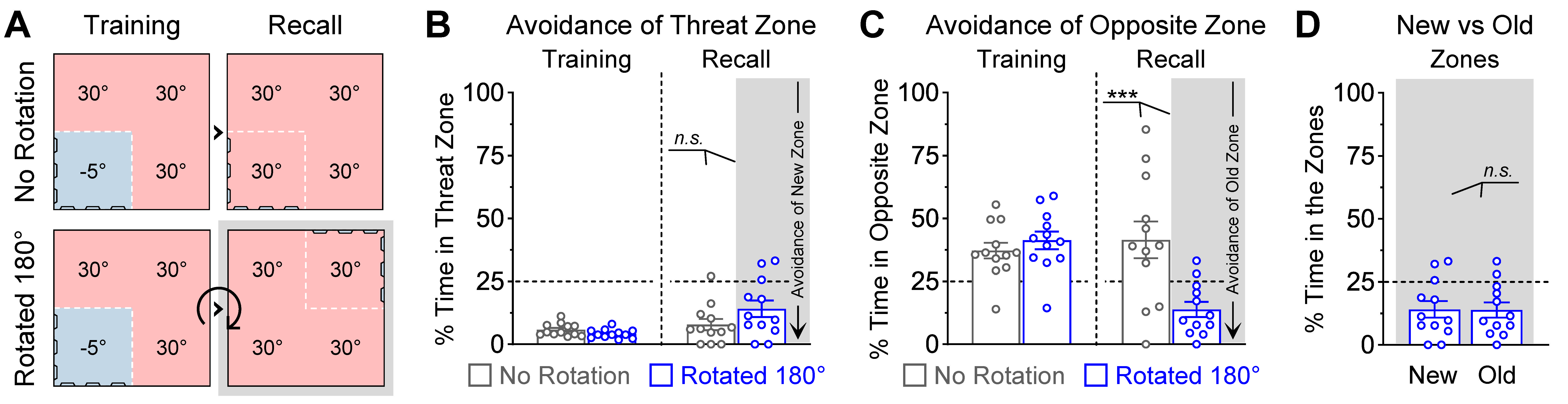

Supplement: Figure 1-2 — Visuospatial cues contributed to memory formation during the thermal threat task. A, Experimental design. For the experimental group, the box was rotated 180° during the recall test, so that the visual cues that predicted threat were now positioned somewhere else (No Rotation: N = 12, Rotated 180°: N = 12). B, Avoidance of the zone that predicted thermal threat, relative to the position of the visual cues. Similar to controls, the experimental group exhibited robust avoidance to the correct zone that predicted threat (Group, F(1,22) = 1.24, P = 0.28; Time, F(1,22) = 9.16, P = 0.0062; Interaction, F(1,22) = 4.00, P = 0.058; Training, P > 0.99; Recall, P = 0.070). C, Avoidance of the opposite zone. Interestingly, the experimental group exhibited robust avoidance to this zone, which was the one associated with thermal threat during the training session. A significant group difference was detected during recall (Group, F(1,22) = 7.37, P = 0.013; Time, F(1,22) = 5.61, P = 0.027; Interaction, F(1,22) = 10.6, P = 0.0037; Training, P > 0.99; Recall, ***P = 0.0002). D, A comparison of avoidance behavior during the recall test, considering the new location versus the old location of the visual cues that predicted the threat zone. The group with rotated cues exhibited similar levels of avoidance to the new and old locations (Paired T-test: t(11) = 0.06, P = 0.96). Therefore, while these findings are consistent with the idea that proximal cues played an important role for the integration of spatial and thermal information to promote learning for the threat zone, these findings also suggest that distal cues (e.g., other visual elements within the procedure room) may have also played a significant role. Download Figure 1-2, TIF file. [file eneuro-11-ENEURO.0140-23.2024-s006.tif]

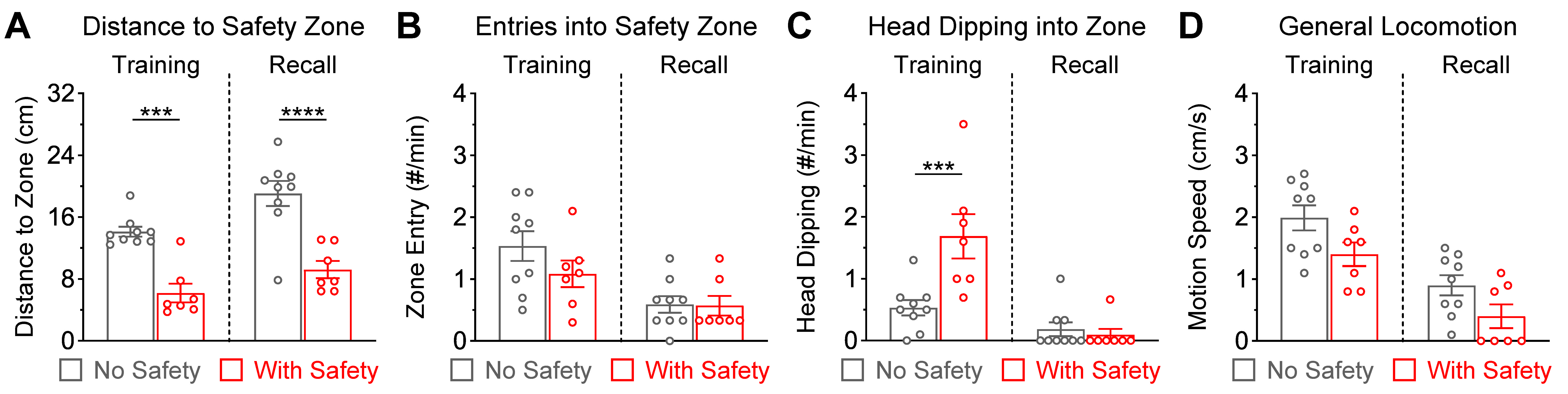

Supplement: Figure 2-1 — Additional measurements during the thermal safety task. A, Average distance to the safety zone, measured from the center of the mouse body to the center of the zone (Group, F(1,14) = 59.9, P < 0.0001; Time, F(1,14) = 9.19, P = 0.009; Interaction, F(1,14) = 0.52, P = 0.48; Training: ***P = 0.0002; Recall: ****P < 0.0001). B, Entries into the safety zone (Group, F(1,14) = 1.34, P = 0.27; Time, F(1,14) = 14.9, P = 0.002; Interaction, F(1,14) = 1.28, P = 0.27; Training: P = 0.23; Recall: P > 0.99). C, Head dipping into the safety zone (Group, F(1,14) = 6.46, P = 0.024; Time, F(1,14) = 34.0, P < 0.0001; Interaction, F(1,14) = 14.0, P = 0.002; Training: ***P = 0.0004; Recall: P > 0.99). D, General locomotion in the arena (Group, F(1,14) = 6.29, P = 0.025; Time, F(1,14) = 43.3, P < 0.0001; Interaction, F(1,14) = 0.08, P = 0.78; Training: P = 0.074; Recall: P = 0.15). [No Safety: N = 9, With Threat: N = 7]. Download Figure 2-1, TIF file. [file eneuro-11-ENEURO.0140-23.2024-s007.tif]

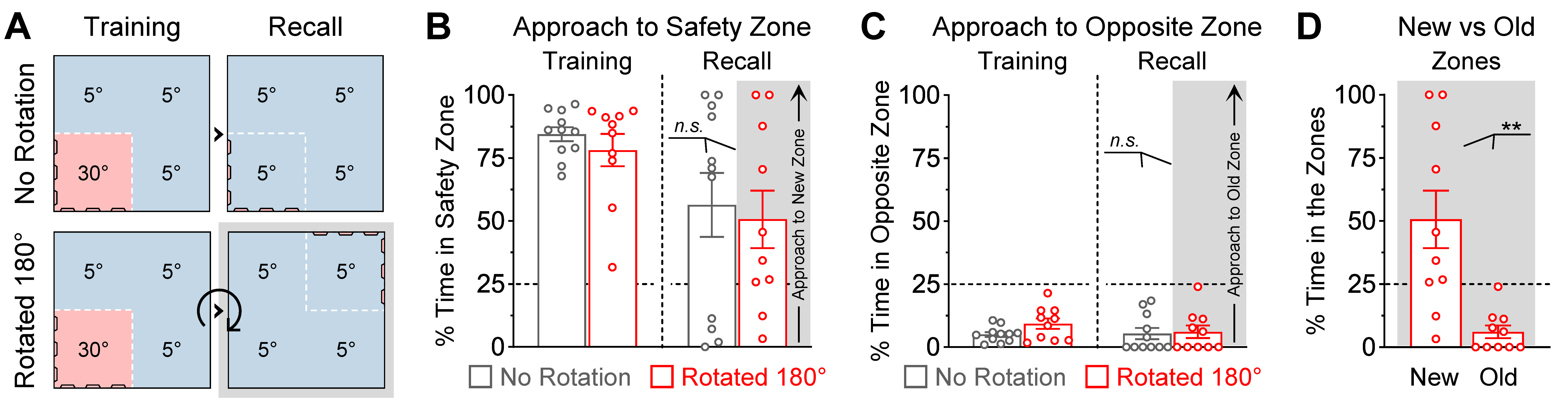

Supplement: Figure 2-2 — Visuospatial cues contributed to memory formation during the thermal threat task. A, Experimental design. For the experimental group, the box was rotated 180° during the recall test, so that the visual cues that predicted threat were now positioned somewhere else (No Rotation: N = 11, Rotated 180°: N = 10). B, Approach to the zone that predicted thermal safety, relative to the position of the visual cues. Similar to controls, the experimental group exhibited approach behavior to the zone that correctly predicted safety (Group, F(1,19) = 0.36, P = 0.56; Time, F(1,19) = 11.1, P = 0.0035; Interaction, F(1,19) = 0.001, P = 0.97; Training, P > 0.99; Recall, P > 0.99). C, Approach to the opposite zone. Both groups exhibited low levels of approach behavior to this zone (Group, F(1,19) = 1.20, P = 0.29; Time, F(1,19) = 0.67, P = 0.42; Interaction, F(1,19) = 1.11, P = 0.30; Training, P = 0.28; Recall, P > 0.99). D, A comparison of approach behavior during the recall test, considering the new location versus the old location of the visual cues that predicted the safety zone. The group with rotated cues exhibited significantly more approach to the new location (Paired T-test: t(9) = 3.41, **P = 0.008). These findings are consistent with the idea that proximal visual cues played a prominent role for the integration of spatial and thermal information to promote learning for the safety zone. Download Figure 2-2, TIF file. [file eneuro-11-ENEURO.0140-23.2024-s008.tif]

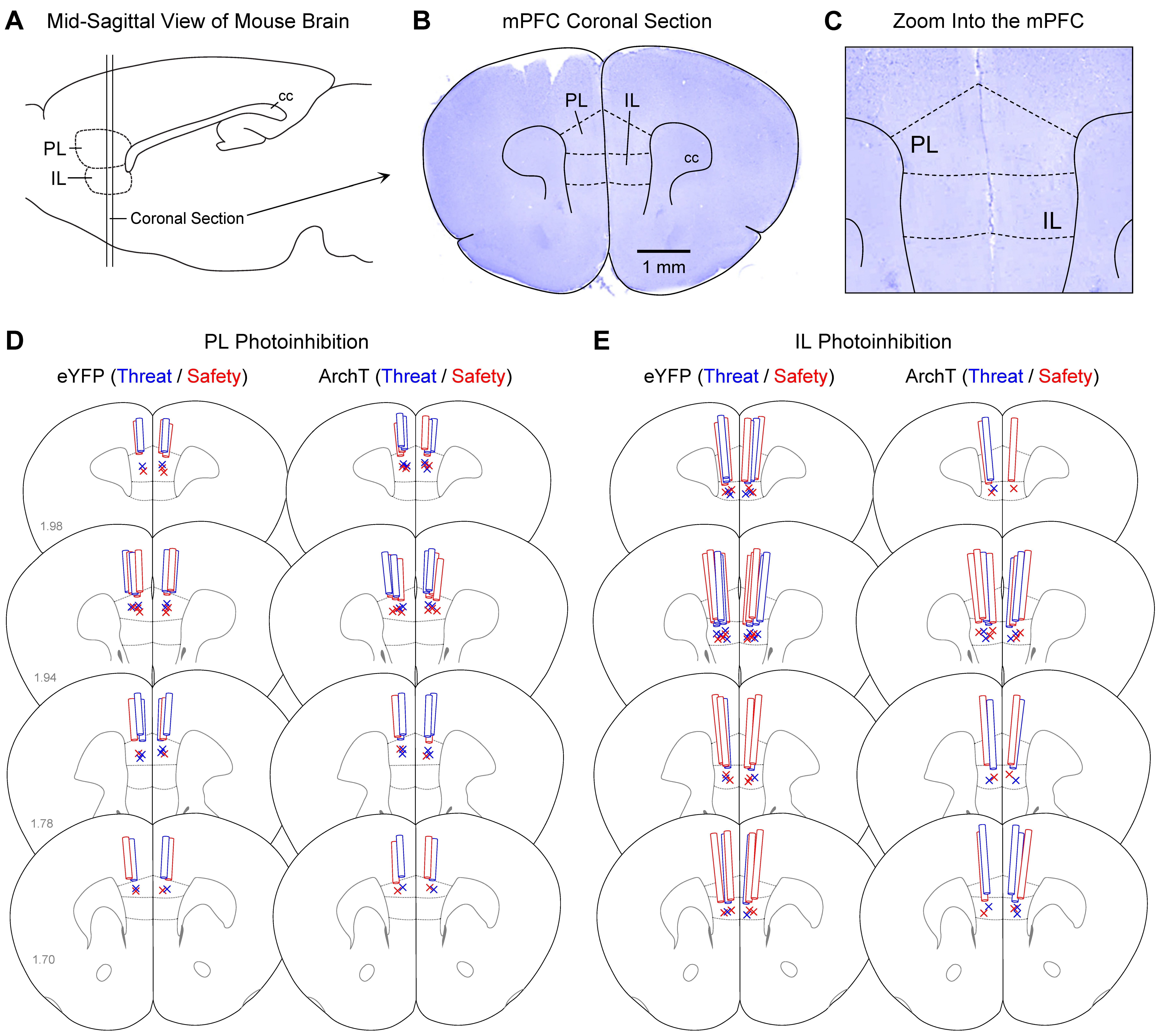

Supplement: Figure 3-1 — Reconstruction of the optical fiber placements and the sites of viral infusions for the optogenetic experiments in Figure 3. A, Mid-sagittal drawing of the mouse brain illustrating the PL and IL regions of the mPFC. B-C, Coronal photomicrographs of the mPFC. Tissue landmarks and key cytoarchitectonic features of the mPFC (e.g., transitions in the cortical layers) were carefully examined in each mouse to separate the PL and IL regions, and to determine whether there was appropriate bilateral targeting of the individual brain regions. Mice were excluded if the individual targets were missed with the optical fibers or if there was viral leakage. D-E, Summary of the optical fiber placements (represented with colored tubes) and viral infusion centroids (represented with colored “×” symbols). Numbers within the coronal drawings indicate the anterior-posterior coordinates in millimeters relative to bregma. [eYFP: control groups, ArchT: neural inhibition groups, cc: corpus callosum]. Download Figure 3-1, TIF file. [file eneuro-11-ENEURO.0140-23.2024-s009.tif]

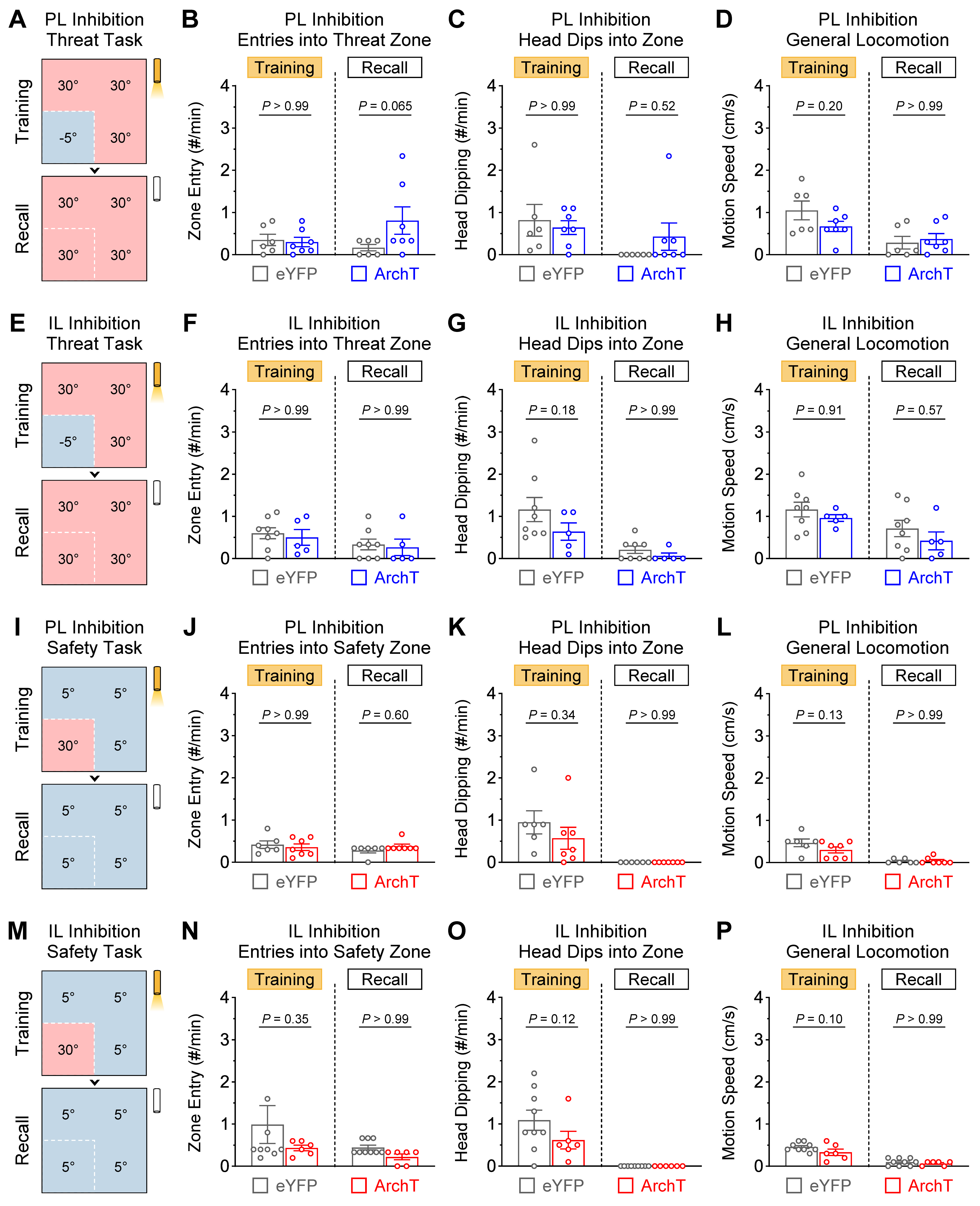

Supplement: Figure 3-2 — PL and IL inhibition did not affect other behavioral measurements during the thermal tasks. A-D, During the threat task, PL inhibition did not affect the rate of entries into the threat zone, head dipping into the threat zone, or general locomotion within the entire test apparatus (eYFP: N = 6, ArchT: N = 7). E-H, During the threat task, IL inhibition did not affect any of these behavioral measurements either (eYFP: N = 8, ArchT: N = 5). I-L, During the safety task, PL inhibition did not produce any significant effects on these behavioral measurements (eYFP: N = 6, ArchT: N = 7). M-P, During the safety task, IL inhibition did not affect these behavioral measurements either (eYFP: N = 9, ArchT: N = 6). [P-values represent Bonferroni post hoc tests]. Download Figure 3-2, TIF file. [file eneuro-11-ENEURO.0140-23.2024-s010.tif]

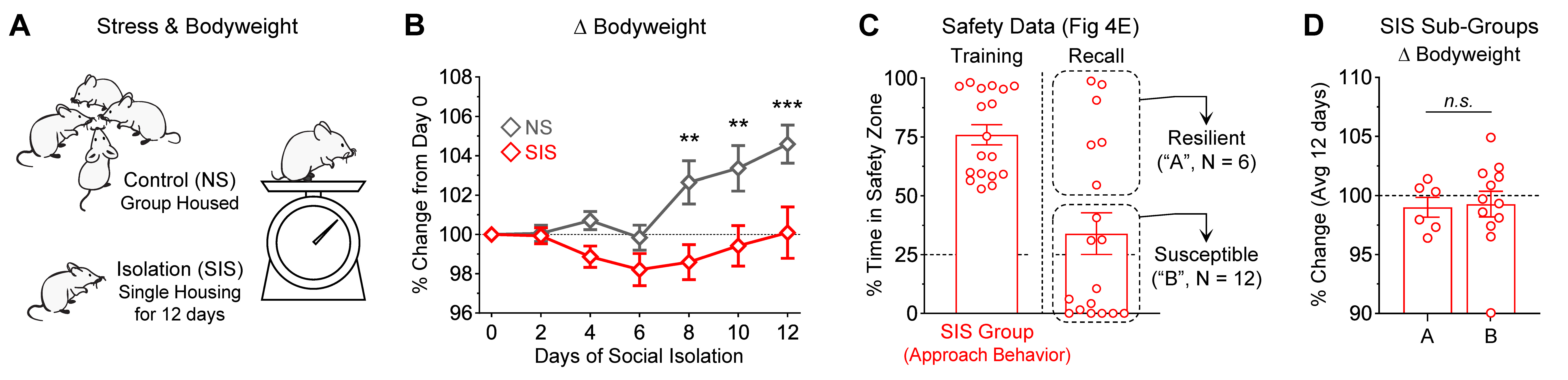

Supplement: Figure 4-1 — Stress-induced alterations in bodyweight. A, Social isolation stress was performed for twelve days, and bodyweights were measured every other day. B, Compared to no-stress controls (NS = 18), mice that underwent social isolation stress (SIS = 18) showed a significant impairment in weight gain (Group, F(1,34) = 7.57, P = 0.009; Time, F(6,204) = 7.11, P < 0.0001; Interaction, F(6,204) = 5.13, P < 0.0001; **P < 0.01, ***P < 0.001). C, Stressed mice were separated into two sub-groups (“Stress Resilient” vs “Stress Susceptible”) based on whether they showed or did not show deficits during the thermal safety task. A cutoff was arbitrarily set at 50%, based on time spent in the safety zone during the recall test. D, No significant differences were detected in bodyweight between the resilient and susceptible groups (Unpaired T-test: P = 0.87). Download Figure 4-1, TIF file. [file eneuro-11-ENEURO.0140-23.2024-s011.tif]

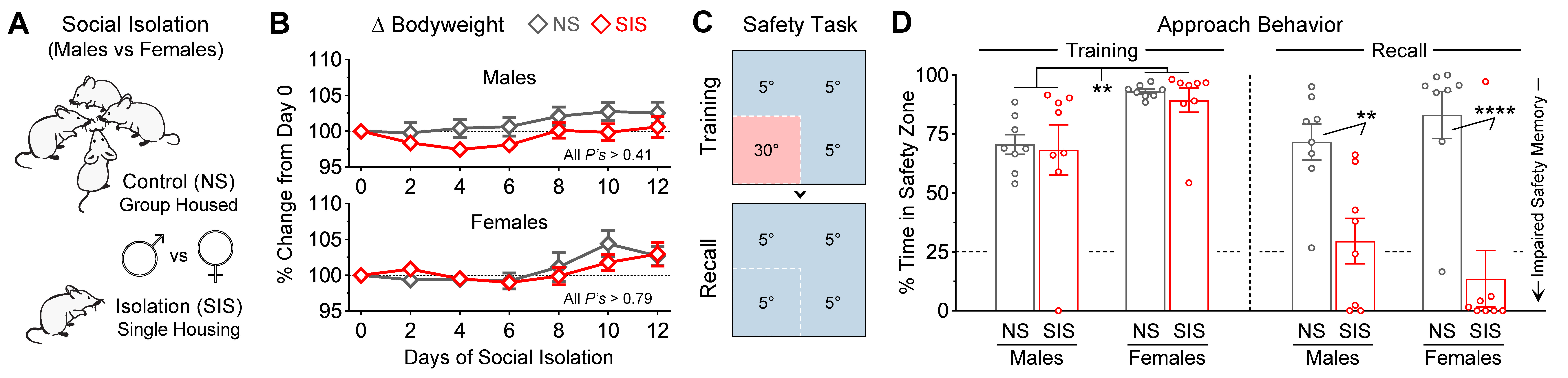

Supplement: Figure 4-2 — Evaluation of potential sex differences in stress vulnerability during the thermal safety task. A, Social isolation stress was performed for twelve days in new cohorts of male and female mice. Male-NoStress (M-NS, N = 8), Male-Stress (M-SIS, N = 8), Female-NoStress (F-NS, N = 8), and Female-Stress (F-SIS, N = 8). B, Changes in bodyweight across the stress days compared to controls (Males: Group, F(1,14) = 2.29, P = 0.15; Time, F(6,84) = 5.86, P < 0.0001; Interaction, F(6,84) = 1.24, P = 0.30) (Females: Group, F(1,14) = 0.06, P = 0.81; Time, F(6,84) = 8.52, P < 0.0001; Interaction, F(6,84) = 1.39, P = 0.23). C, Behavioral testing in the thermal safety task was conducted twelve days after the cessation of stress. D, Approach behavior assessed as the time spent within the safety zone (Group, F(3,28) = 8.04, P = 0.0005; Time, F(1,28) = 34.4, P < 0.0001; Interaction, F(3,28) = 10.7, P < 0.0001). Overall, females exhibited more time within the safety zone than males during the training session (all males versus all females, **P = 0.0026). Such sex difference disappeared during the long-term recall test (all males versus all females, P > 0.99). Yet, the stressed groups in both males and females exhibited significantly less time within the zone that predicted safety during the recall test (**P = 0.009, ****P < 0.0001), suggesting that stress impaired safety memory formation in a sex-independent manner. Download Figure 4-2, TIF file. [file eneuro-11-ENEURO.0140-23.2024-s012.tif]

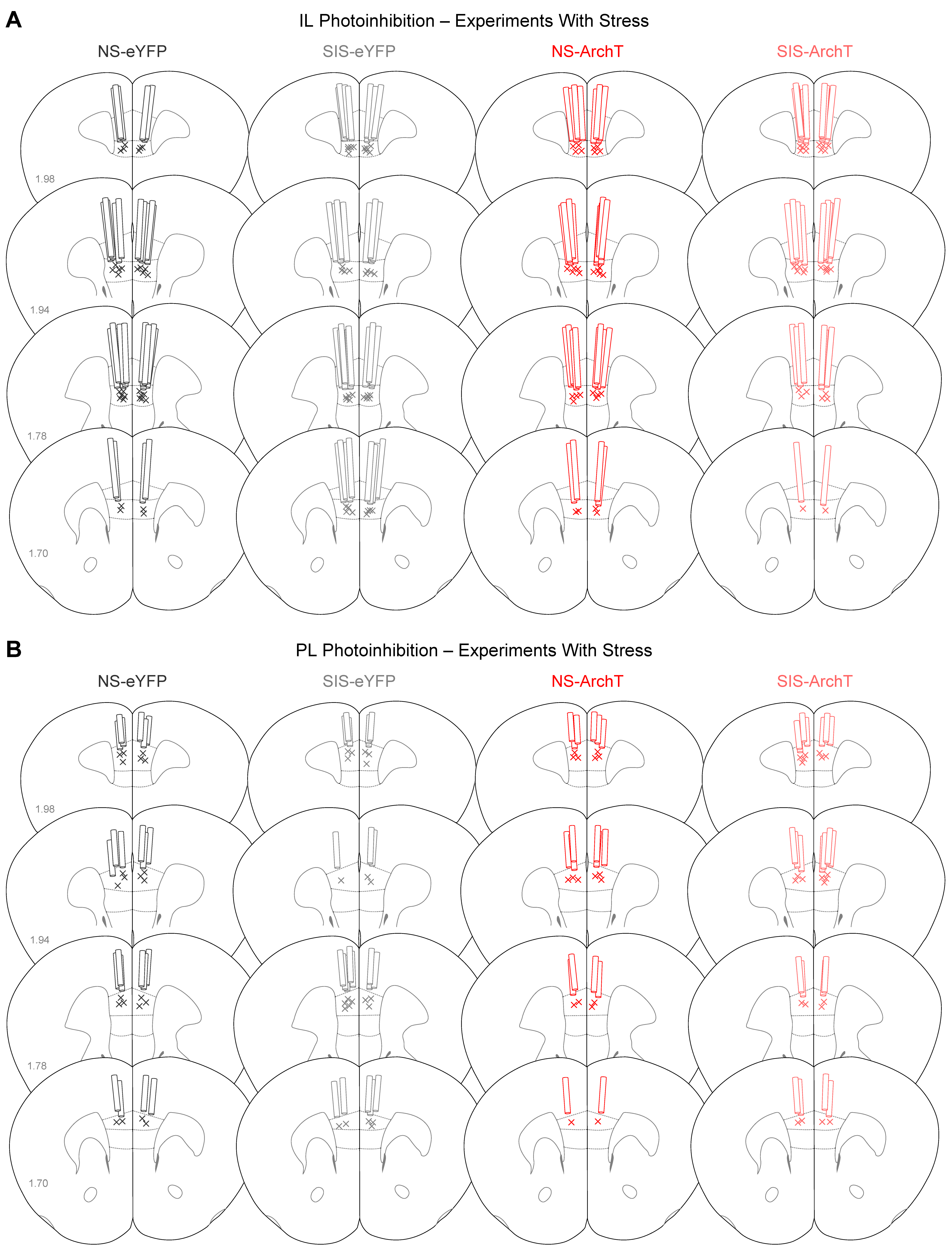

Supplement: Figure 5-1 — Fiber placements and viral infusion sites for the optogenetic experiments that also included stress treatments in Figure 5. A, Summary of IL targeting. B, Summary of PL targeting. The colored tubes represent the optical fibers, while the “×” symbols represent the viral infusion sites. [NS: no stress, SIS: social isolation stress, eYFP: control fluorophore, ArchT: neural inhibition opsin]. Download Figure 5-1, TIF file. [file eneuro-11-ENEURO.0140-23.2024-s013.tif]

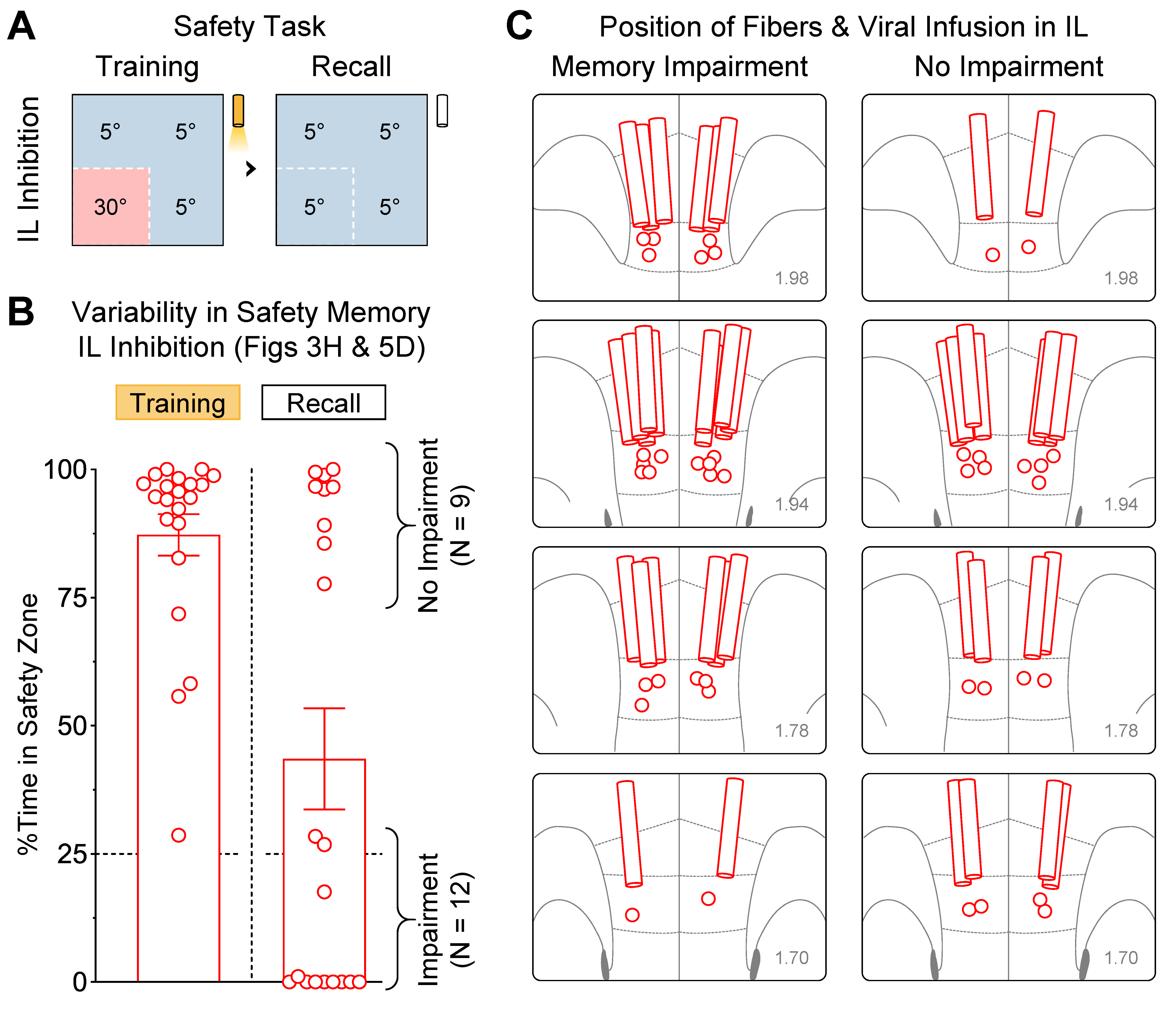

Supplement: Figure 5-2 — Individual variability in the effects of IL inhibition during the thermal safety task was unrelated to variability in the position of optical fibers or location of viral infusions. A, Schematic of the safety task with IL inhibition occurring during the training session. B, Combined IL inhibition data from Fig 3H (ArchT group) and Fig 5D (NS-ArchT group). Mice were bimodally distributed, with some exhibiting robust impairment in safety memory (12/21, 57%), and others exhibiting no impairment (9/21, 43%). C, Coronal drawings illustrate the position of optical fibers and viral infusion sites in IL. No major differences were appreciated between these two sub-groups. IL targeting seemed similarly distributed across the anterior-posterior, medial-lateral, and dorsal-ventral planes in the two groups. Download Figure 5-2, TIF file. [file eneuro-11-ENEURO.0140-23.2024-s014.tif]

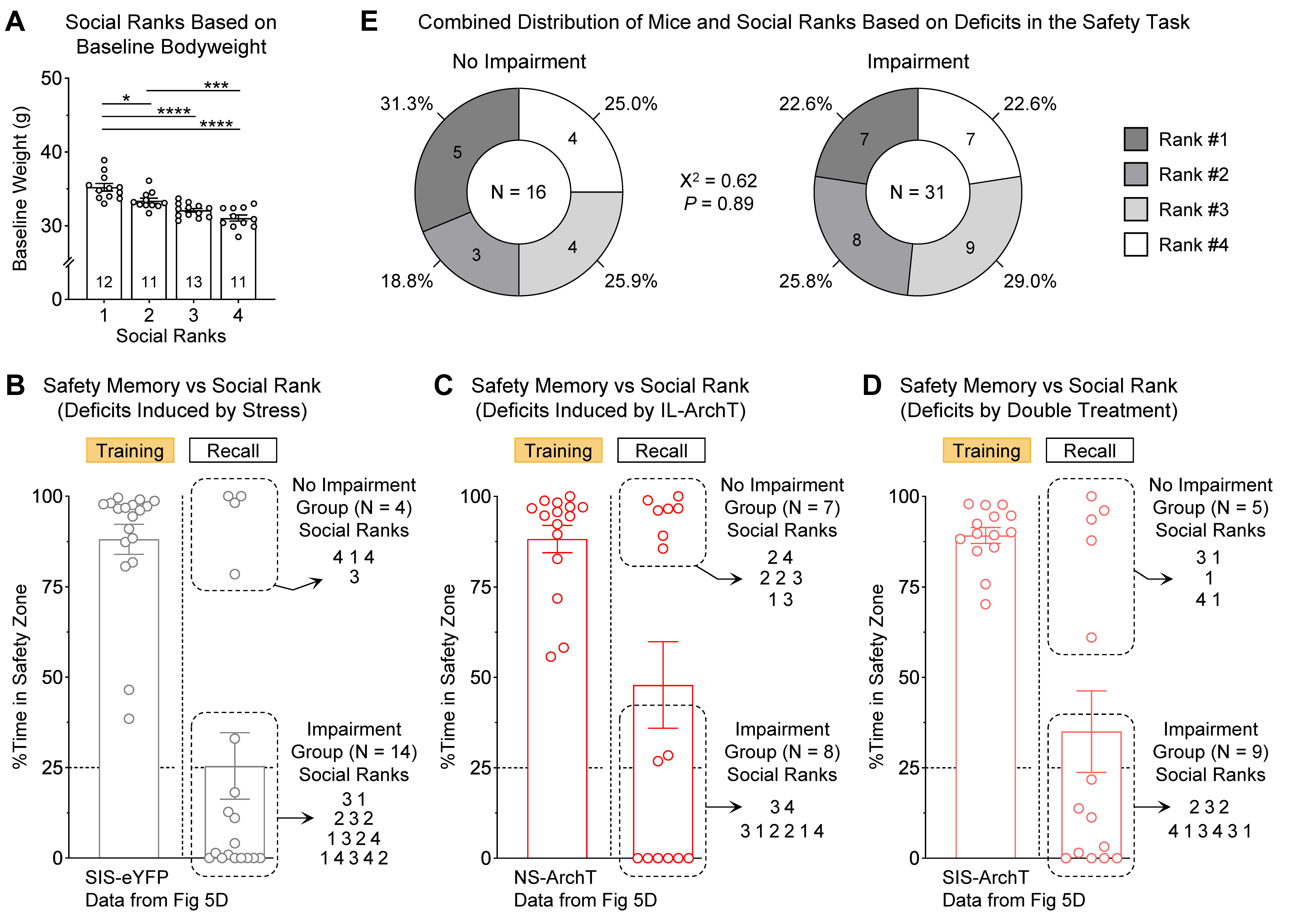

Supplement: Figure 5-3 — Social status did not contribute to individual variability produced by either stress or infralimbic inhibition during the thermal safety task. A, Within each homecage, mice were assigned social ranks based on differences in bodyweight, measured prior to any treatment or behavioral testing. Digits within the bars indicate the number of mice per rank (One-way ANOVA: F(3,43) = 21.3, P < 0.0001; Bonferroni Tests: *P = 0.013, ***P = 0.0009, ****P < 0.0001). B-D, Mice were further separated into sub-groups based on whether they showed or did not show impairment in safety memory during the safety task. B, In mice that received the SIS-eYFP treatment (i.e., stress but no photoinhibition) social ranks seemed similarly distributed between the no-impairment and impairment groups. C, In mice that received the NS-ArchT treatment (i.e., IL photoinhibition but no stress), social ranks also seemed similarly distributed between the two groups. D, Finally, in mice that received the SIS-ArchT double treatment (i.e., stress and IL photoinhibition), social ranks also seemed similarly distributed between the no-impairment and impairment groups. E, Combined distributions, based on social ranks and deficits in the safety task. No significant difference was detected based on a chi-square test. Download Figure 5-3, TIF file. [file eneuro-11-ENEURO.0140-23.2024-s015.tif]
